# Evaluation of distinct molecular architectures and coordinated regulation of the catabolic pathways of oestrogenic dioctyl phthalate isomers in *Gordonia* sp.

**DOI:** 10.1099/mic.0.001353

**Published:** 2023-06-29

**Authors:** Rinita Dhar, Suman Basu, Mousumi Bhattacharyya, Tapan K. Dutta

**Affiliations:** ^1^​ Department of Microbiology, Bose Institute, EN-80, Sector V, Salt Lake, Kolkata – 700091, West Bengal, India

**Keywords:** biodegradation, di(2-ethylhexyl) phthalate, di-*n*-octyl phthalate, *Gordonia*, multi-omics analysis, phthalic acid esters

## Abstract

Bacterial strain GONU, belonging to the genus *Gordonia *, was isolated from a municipal waste-contaminated soil sample and was capable of utilizing an array of endocrine-disrupting phthalate diesters, including di-*n*-octyl phthalate (DnOP) and its isomer di(2-ethylhexyl) phthalate (DEHP), as the sole carbon and energy sources. The biochemical pathways of the degradation of DnOP and DEHP were evaluated in strain GONU by using a combination of various chromatographic, spectrometric and enzymatic analyses. Further, the upregulation of three different esterases (*estG2*, *estG3* and *estG5*), a phthalic acid (PA)-metabolizing *pht* operon and a protocatechuic acid (PCA)-metabolizing *pca* operon were revealed based on *de novo* whole genome sequence information and substrate-induced protein profiling by LC-ESI-MS/MS analysis followed by differential gene expression by real-time PCR. Subsequently, functional characterization of the differentially upregulated esterases on the inducible hydrolytic metabolism of DnOP and DEHP revealed that EstG5 is involved in the hydrolysis of DnOP to PA, whereas EstG2 and EstG3 are involved in the metabolism of DEHP to PA. Finally, gene knockout experiments further validated the role of EstG2 and EstG5, and the present study deciphered the inducible regulation of the specific genes and operons in the assimilation of DOP isomers.

## Graphical Abstract

Biochemical and molecular analyses to reveal the regulation of catabolic pathways in the complete degradation of dioctyl phthalate isomers in *Gordonia* sp.
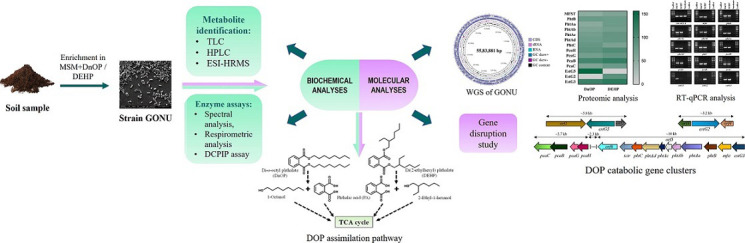

## Introduction

Phthalate esters or phthalic acid esters (PAEs), commonly known as phthalates, are the predominant plasticizers used industrially in a broad range of consumer products because of their exclusive properties of enhancing product durability, glossiness, longevity and flexibility [1]. PAEs account for 70 % of the global plasticizer market, and more than eight million tonnes of PAEs are consumed per year worldwide [2, 3]. PAEs are non-covalently bound to the polymer matrix and can slowly migrate into the environment from host polymers, eventually entering the food chain and causing bio-magnification [4, 5]. Nonetheless, PAE vulnerability occurs through skin contact, breathing, and food or water consumption [6]. PAEs are known as endocrine-disrupting chemicals as they interfere with the biological activity of the endocrine system by hormone mimicry and blocking or altering hormone metabolism. Due to their multiple adverse health effects, some phthalates were restricted in children’s toys by the European Union and the US Environmental Protection Agency (USEPA: https://www.epa.gov/sites/default/files/2015-09/documents/phthalates_actionplan_revised_2012-03-14.pdf). Nevertheless, considerable global attention is warranted primarily for their oestrogenic properties [7, 8]. Among the phthalate diesters, dimethyl phthalate (DMP), diethyl phthalate (DEP), di-*n*-butyl phthalate (DBP), di-*n*-octyl phthalate (DnOP), benzyl butyl phthalate (BBP) and di(2-ethylhexyl) phthalate (DEHP) have already been listed as priority environmental pollutants by the USEPA [9].

Since the natural processes of PAE degradation occur on indefinite time scales, a directed process of biodegradation is being initiated and employed as an alternative strategy for the elimination of PAEs to avoid its significant route of natural dissipation in different environments [5, 6, 10]. Numerous studies have revealed that bacteria, fungi, algae and even yeasts can degrade PAEs under aerobic and anaerobic conditions [11, 12]. Because of their hydrophobic nature and low susceptibility to biodegradation, high-molecular-weight (HMW) phthalates (BBP, DEHP and DnOP) were identified as priority pollutants [13]. To date, many PAE-degrading bacterial strains have been characterized; however, the majority of these strains were reported to degrade low-molecular-weight phthalates [14–18].

**Fig. 1.**
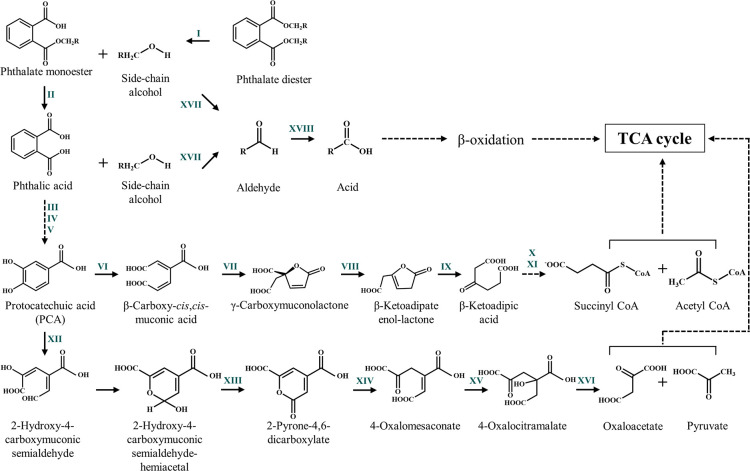
Conventional bacterial metabolic pathway for the complete degradation of phthalic acid diesters. Pathway enzymes: I, esterase; II, esterase; III, phthalate 3,4-dioxygenase/phthalate-4,5-dioxygenase; IV, *cis*-3,4-dihydroxy-3,4-dihydrophthalate dehydrogenase/*cis*-4,5-dihydroxy-4,5-dihydrophthalate dehydrogenase; V, 3,4-dihydroxyphthalate-2-decarboxylase/4,5-dihydroxyphthalate-2-decarboxylase; VI, protocatechuate 3,4-dioxygenase; VII, β-carboxymuconate cycloisomerase; VIII, γ-carboxymuconolactone decarboxylase; IX, β-ketoadipate enol-lactone hydrolase; X, β-ketoadipate succinyl-CoA transferase; XI, β-ketoadipyl CoA thiolase; XII, protocatechuate 4,5-dioxygenase; XIII, 2-hydroxy-4-carboxymuconic semialdehyde dehydrogenase; XIV, 2-pyrone-4,6-dicarboxylate hydrolase; XV, 4-oxalomesaconate hydratase; XVI, 4-oxalocitramalate aldolase; XVII; NAD(P)^+^-dependent alcohol dehydrogenase; XVIII, NAD(P)^+^-dependent aldehyde dehydrogenase.

Nonetheless, in the microbial degradation of a wide range of PAEs, several studies have evaluated metabolic pathways and degradation kinetics, deciphering the involvement of a hydrolase, the first enzyme to initiate the metabolic processes [19–23]. PAE-hydrolysed aliphatic or aromatic side chain alcohols and phthalic acid are generally metabolized, leading to the TCA cycle via the central pathway and allowing the organism to grow. A general scheme for the bacterial PAE degradation pathway is depicted in Fig. 1. Until recently, 60 bacterial strains were reported to degrade various PAEs, of which a few distinctive genera, namely *Gordonia *, *Sphingomonas *, *Pseudomonas* and *Rhodococcus,* are prevalent [3].

Due to the poor rate of abiotic transformation of HMW PAEs, the metabolic breakdown of DnOP and DEHP is considered one of the best possible abatement alternatives [24]. There have been fewer reports on the biodegradation of DnOP and DEHP than on short-alkyl-chain PAEs. All these studies primarily revealed the biodegradation potential of an individual isolate or consortium. Only a few illustrated metabolic pathways at the biochemical level and quite a few documented probable catabolic genes based on whole genome sequence information [25, 26]. However, there are few informative resources for experimentally validated molecular data on the nature of esterase(s) and other catabolic genes involved in the complete assimilation of DnOP or DEHP.

In the present study, we describe the isolation of a bacterial strain capable of degrading an array of PAEs, including DnOP and its isomer DEHP. The inducible metabolic pathways for the assimilation of the dioctyl phthalate (DOP) isomers were appraised biochemically, followed by the detection of the relevant genes from whole genome sequences and evaluation of substrate-specific catabolic modules via differential protein and RNA expression studies. Consequently, the present study allowed us to characterize the regulation of three different types of esterases: diesterase-monoesterase, diesterase and monoesterase, validated by *in vivo* and *in vitro* analyses using both wild-type and gene knockout mutant strains.

## Methods

### Chemicals and kits

DMP, DEP, DBP, BBP, DEHP, DnOP, mono(2-ethylhexyl) phthalate (MEHP), 1-octanal, 1-octanal, 1-octanoic acid, 2-ethyl-1-hexanol, 2-ethyl-1-hexanal, 2-ethyl-1-hexanoic acid, phthalic acid (PA) and protocatechuic acid (PCA) were purchased from Sigma-Aldrich. Mono-*n*-octyl phthalate (MnOP) was procured from Chempure. All other chemicals and solvents used in the present study were of analytical or HPLC grade and employed without further purification.

### Enrichment, isolation and characterization of PAE-assimilating bacterial strain

A municipal waste-contaminated soil sample was used for the enrichment of culture in a liquid mineral salt medium (MSM [27]), supplemented with DnOP (0.5 g l^−1^) as the sole source of carbon and energy, and incubated at 28 °C on a rotary shaker (180 r.p.m.). Through several rounds of sub-culturing in liquid medium and subsequent plating on Luria-Bertani agar medium containing 1.8 % agar-agar (Hi-Media), a DnOP-degrading pure culture was isolated. For characterization of the isolate, designated as strain GONU, a combination of universal primers f27 and r1492 [28] was used to amplify the 16S rRNA gene. The gene was sequenced following the manufacturer’s instructions for *Taq* DNA polymerase-initiated cycle sequencing reactions using fluorescently labelled di-deoxynucleotide terminators with an ABI PRISM 377 automated sequencer (Perkin–Elmer Applied Biosystems). The 16S rRNA sequence was subjected to a homology search in blast (http://www.ncbi.nlm.nih.gov/BLAST) to evaluate the phylogenetic affiliation of strain GONU. Then, the 16S rRNA gene sequences of the top 50 closest type strains were collected from GenBank, and aligned (ClustalX2), and a phylogenetic tree was reconstructed using the neighbour-joining method [29]. The tree was visualized and labelled in Tree Explorer v2.12.

### Genome sequencing, assembly and annotation

Genomic DNA was isolated from the Luria Bertani broth-grown culture (24 h at 28 °C) of strain GONU using the PureLink Genomic DNA Kit (Invitrogen) according to the manufacturer’s protocol. The purified genomic DNA was used for *de novo* whole genome sequencing on an Illumina NovaSeq 6000 platform using the paired-end strategy. The genome was annotated by the DFAST tool, which took scaffolds of the desired length (scaffolds shorter than 500 bp in length were discarded). Analysis of Clusters of Orthologous Groups (COGs) of proteins was performed on the prokaryotic protein database using Reverse Position-Specific blast (RPS blast using NCBI COG version 2/2/2011) with an e-value of 0.001; data were plotted using GraphPad Prism v8.0.1. Visualization of the circular genome of strain GONU was derived from the web server Proksee (https://beta.proksee.ca/).

### Culture conditions and isolation of metabolic intermediates

Strain GONU, a DnOP-degrading bacterial isolate, was cultured in 100 ml Erlenmeyer flasks containing 25 ml MSM and 0.5 g l^−1^ DnOP, DEHP, MnOP, MEHP, 1-octanol, 1-octanal, 1-octanoic acid, 2-ethyl-1-hexanol, 2-ethyl-1-hexanol, 2-ethyl-1-hexanoic acid, PA, PCA or succinate individually as the sole carbon and energy sources. The cultures were incubated at 28 °C on a rotary shaker (180 r.p.m.). Other PAEs, such as DMP, DEP, DBP or BBP, were also used as growth substrates under identical culture conditions to evaluate substrate utilization potential. Monitoring the OD_600 nm_ of culture media was used to account for growth.

For resting cell transformation, a late log-phase culture grown on specific PAE was harvested by centrifugation (8000 *g* for 15 min), washed three times with 50 mM potassium phosphate buffer (PPB, pH 7.0), and resuspended in the same buffer to attain an OD_600_ of 1.0. Then, the washed cell suspensions were incubated individually with DnOP, DEHP or their possible metabolic intermediates for various time points. After incubation, the reaction mixtures were extracted three times with an equal volume of ethyl acetate before and after acidification with 6 M HCl to a pH of 1.5–2.0. The combined organic extracts were evaporated in a rotary evaporator (R-100; BUCHI) under reduced pressure. The residues were dissolved in 1 ml of HPLC-grade ethyl acetate, syringe filtered using a 0.22 µm nylon membrane and stored in HPLC vials (Borosil; pre-rinsed with 30 % nitric acid followed by 2 M ammonium hydroxide) for further analysis. Unless stated otherwise, all experiments were done in triplicate with parallel culture controls.

To understand cell morphology, DnOP and succinate-grown log-phase cultures were harvested as above, and the cells were mounted on slides, fixed with 2.5 % glutaraldehyde at 4 °C for 1 h, and then dehydrated with a series of ethanol solutions (30, 50, 70, 80, 90 and 100 %, v/v). Samples were gold-coated and visualized under a scanning electron microscope (FEI Quanta 200).

### Preparation of cell-free extract

Washed late log-phase culture cell suspensions were individually loaded into a pre-cooled Constant Cell Disruption System, One Shot Model (Constant System), attached with an 8.0 ml capacity chamber. The cell suspension was lysed at 30 000 psi for two cycles, followed by centrifugation (13 000 *g* for 30 min) at 4 °C. The supernatant was used to provide cell-free enzymes for additional biochemical and proteomic research. Protein was measured by the method of Lowry *et al.* [30], with BSA as the standard.

### Respirometric investigation

Consumption of molecular oxygen by washed cell suspension in the presence of substrate or a possible intermediate was assayed at 25 °C with a YSI Model 5300A biological oxygen monitor (Yellow Springs Instrument) equipped with a Clark-type polarographic oxygen electrode (YSI-model 5331A oxygen probe). The reaction mixture contained 200 µl of cell suspension with an OD_600_ of 1.0 (25 mg cells, wet weight), 500 µl of the substrate and 2.3 ml PPB (50 mM, pH 7.0) in a total volume of 3.0 ml. The reaction was started by adding assay substrate (water saturated), and oxygen uptake was monitored continuously for 10 min. The oxygen uptake rate was expressed in nmol min^−1^ (mg protein)^−1^. The rates were corrected for cellular respiration or endogenous oxygen consumption.

### Chromatographic and spectrometric analyses

To reveal metabolic pathways, the organic extract of spent culture containing residual DOP isomers and their metabolites were resolved by TLC on silica gel 60 GF_254_ plates (Merck) using the solvent system hexane/chloroform/glacial acetic acid (10 : 3 : 2) and detected by a UV lamp at 254 nm. The resolved products were identified by comparing the authentic standards, and processed identically. Further, the organic extracts were analysed by HPLC using a Shimadzu model LC20-AT pump system (Shimadzu) fitted with a model SIL-M20A diode array detector and an analytical Agilent C18 reverses-phase column equipped with a model SIL-20A auto-sampler. The metabolites and the unconverted substrate were eluted using the mobile phase of methanol (solvent A) and water (pH 3.0) (solvent B) using a programmed gradient of 30 % A – 70 % B (0–8 min), 50 % A – 50 % B (10–25 min), 95 % A – 5 % B (25–53 min), 50 % A – 50 % B (53–55 min), 30 % A – 70 % B (55–58 min). The flow rate was set to 1.0 ml min^−1^, and the eluted compounds were detected at 254 nm. Metabolic intermediates were verified by comparing their retention times and UV-visible spectra (obtained from diode array analysis) with those of the reference compounds analysed under the same set of conditions. Again, to confirm the metabolic profiles, the organic extracts were analysed by employing direct infusion electron spray ionization high-resolution MS (DI-ESI-HRMS) using a mass spectrometer (Xevo-G2-Xs-Q TOF; Waters). Data were accumulated in positive and/or negative ion mode, and the full scan mass spectra were obtained over a mass range of *m/z* 50–500. The capillary voltage was set at 3000 V, and the source and desolvation temperatures were 80 and 250 °C, respectively. The cone gas flow rate was set to 50 l h^−1^. The molecular weight (*m/z*) of the biodegraded metabolites was confirmed by comparing the *m/z* values of respective authentic standards analysed under identical conditions.

### Enzyme assays

#### Oxidase assay

To determine oxidase activity, a 2,6-dichlorophenol indophenol (DCPIP) reduction assay was performed spectrophotometrically, as described earlier [31]. The individual reaction mixture comprises 25 µl of 6.7 mM DCPIP, 50 µl of 20 mM phenazine methosulfate, 30 µl of the substrate and 50 µg of cell-free enzyme in 80 mM Tris/HCl (pH 8.7) buffer in a final volume of 3 ml. After incubating at 28 °C, the change in colour was investigated. The experiment was done for 1-octanol and 1-octanal, using cell-free extracts of DnOP, MnOP-succinate, 1-octanoic acid and succinate-grown cells, while 2-ethyl-1-hexanol and 2-ethyl-1-hexanal were used as substrates in the presence of cell-free extracts of DEHP, MEHP-succinate, 2-ethyl-1-hexanoic acid and succinate-grown cells. Appropriate controls were used in parallel to detect any false positive results.

#### Spectrophotometric investigation

Cell-free enzyme-mediated transformations were observed by monitoring the changes in the UV-visible spectra of PCA in a Cary 100 Bio UV-Visible Spectrophotometer (Varian Australia) using quartz cuvettes of 1 cm path length. Reaction mixtures were scanned every minute in the 200–500 nm wavelength range for 10 cycles. Varian Cary Win UV Scan application software was used to analyse the data.

### Evaluation of inducible proteins in dioctyl phthalate metabolism

Strain GONU was subcultured several times in MSM supplemented with DnOP and DEHP to obtain optimized inducible cultures, while succinate-grown cultures were used as a negative control. For each set, late log-phase cells were harvested (8000 *g* for 15 min) and washed three times with 50 mM PPB (pH 7.0). As described above, the harvested cells were lysed to obtain cell-free extract (CFE) preparations. The enzyme preparations were lyophilized using an Edwards lyophilizer and processed further for trypsin digestion, followed by LC-ESI-MS/MS analysis [32] using a Waters Xero-G2-Xs-QTOF system in positive ion mode. A C18 BEH column (Agilent) was used for separation chemistry using water (A) and acetonitrile (B) supplemented with 0.1 % formic acid as a mobile phase in a gradient mode. The time programme used was as follows: 0–30 min, 5 % B; 30–45 min, 35 % B; 45–50 min, 5 % B; and 50–60 min, 0 % B. The flow rate was kept at 30 µl min^−1^. In proteome analysis, the DnOP- and DEHP-inducible peptides were identified based on differential intensities with respect to peptides obtained from succinate-grown cultures. Further, the inducible peptides were matched with whole genome sequence data of strain GONU using the acquisition and processing software Mass Lynx Version 4.1 and ProgenesisQiP, respectively.

### Real-time PCR analysis

The DnOP-, DEHP- and succinate-grown cultures were used for bacterial RNA isolation to verify the DOP-inducible proteome data. Cell lysis was carried out using a Constant Cell Disruption System, One Shot Model, at 30 000 psi for one cycle, followed by the conventional TriZol method for RNA isolation [33]. Before and after DNase treatment (RNase-free DNase I; Thermo Scientific), RNA quality was determined by agarose gel electrophoresis and quantified using a multimode reader (CLARIOstar Plus; BMG Labtech). Samples with an *A*_260_/*A*_280_>1.8 were used for real-time PCR (RT-PCR) analysis. Apart from the DnOP-, DEHP- and succinate-grown cells, the expression of *est*, *pht* and *pca* genes were evaluated by RT-PCR in MnOP-succinate-, MEHP-succinate- and PA-succinate-grown cells. To avoid false-positive RT-PCR results, DNA contamination from isolated RNA was wholly removed until a clear field on agarose gel electrophoresis was expected for the amplified PCR product with RNA as a template. Single-strand cDNA was synthesized using 1 µg RNA (RevertAid First Strand cDNA Synthesis Kit; Thermo Fisher) in the ProFlex PCR system (Applied Biosystems), and RT-PCR was performed in QuantStudio 5 (Applied Biosystems) with 4 µl of 2× DyNAmoColorFlash SYBR Green PCR Master Mix (Thermo Fisher), 2 µl (10 mM) gene-specific RT primers (forward+reverse) (Table S1, available in the online version of this article), cDNA template (1 µl) in a total volume of 10 µl adjusted with molecular biology-grade water (Hi-Media). The 16S rRNA gene was taken as an endogenous control, and amplicon sizes were 200–300 bp for all the genes assessed. Relative gene expression was determined using the ∆∆C_T_ method and visualized by agarose gel electrophoresis.

### Construction of gene deletion mutants

To evaluate the role of DnOP on the inducible catabolic operon(s), the esterase gene responsible for the initial metabolism of DnOP was deleted using the recombination plasmid vector backbone pCM184 with the replacement of *kan*, a kanamycin resistance gene. To facilitate chromosomal site-specific homologous recombination, the homologous upstream (UP) and downstream (DOWN) flanking sequences (321 and 393 bp) of the targeted esterase gene were amplified from the genomic DNA of strain GONU with forward and reverse primers designed for each fragment. Amplified and PCR-purified UP and DOWN fragments were incorporated separately into the *Mlu*I/*Sac*I restriction enzyme cut sites of the vector of MCS2 and the *Eco*RI/*Nde*I sites of MCS1, respectively. Tables S1 and S2 give primer sequences and information of strains and plasmids used in this study, respectively. The esterase deletion mutant strain was constructed by electroporating the recombinant pCM184 plasmid into electrocompetent cells of the wild-type strain GONU [34, 35]. Allelic exchange took place via double recombination of homologous UP and DOWN sites to insert the kanamycin resistance gene cassette from the recombinant pCM184 plasmid into the chromosome of strain GONU by disrupting the esterase gene (Fig. S1). Disruption of esterase and insertion of *kanR* were verified based on PCR amplification and sequencing of the amplicon using an ABI PRISM 377 automated sequencer (Perkin-Elmer Applied Biosystems). Similarly, the other phthalate esterase gene *estG2* involved in DEHP metabolism was successfully disrupted following the protocol mentioned above.

To evaluate the metabolic potential of the esterase deletion mutants, the growth profiles were determined with respect to DnOP, DEHP and their metabolic intermediates individually as sole carbon sources. Further, an induction experiment was performed with the *estG5* deletion mutant, grown in MSM supplemented with succinate plus kanamycin in the presence and absence of DnOP as an inducer molecule. By contrast, for the *ΔestG2* deletion mutant, the strain was grown in MSM supplemented with succinate plus kanamycin in the presence and absence of DEHP as an inducer molecule or in MSM supplemented with kanamycin plus DEHP–MEHP. These cultures were used to determine the gene expression profile of esterases and that of the *pht* and *pca* gene clusters by RT-PCR analysis. Again, to evaluate the role of esterases, the CFE of the DnOP-induced culture of *ΔestG5* was used to check the possible transformation of DnOP and MnOP. Similarly, the CFE of the DEHP-induced culture of the *ΔestG2* mutant was accounted for to check the enzymatic conversion of DEHP and MEHP. All control experiments used succinate plus kanamycin-grown cells of esterase deletion mutants as respective uninduced controls.

## Results

### Isolation and identification of a PAE-transforming bacterium

Restraint enrichment of a municipal waste-contaminated soil sample led to the isolation of strain GONU, which was able to utilize an array of phthalate diesters ranging from C1 to C8 side-chain compounds as a sole source of carbon and energy. Comparative analysis of the 16S rRNA sequence (1524 bp) of the strain using the blast search revealed 99.87 and 98.54% identity (query coverage of 99%) with *Gordonia amicalis* strain IEGM (accession no. NR_028735.1) and *Gordonia desulfuricans* strain 213E (accession no. NR_028734.1), respectively. Based on the phylogenetic analysis depicted in Fig. S2(a), strain GONU was identified as a *Gordonia* sp. belonging to the phylum *Actinomycetota *. Under a scanning electron microscope (6000× magnification), the DnOP- and succinate-grown cells of strain GONU appeared rod-shaped, a characteristic cell morphological feature of the genus *Gordonia *. Nevertheless, the DnOP-grown cells were smaller than those of succinate-grown cells, implying the possible consequence of DnOP-associated stress in the culture medium (Fig. S2b).

### Phthalic acid ester utilization potential

**Fig. 2.**
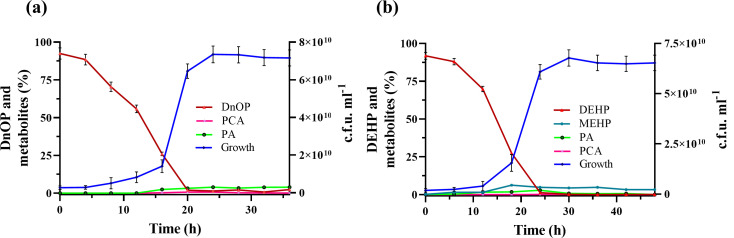
Growth curve and substrate utilization profiles of *Gordonia* sp. strain GONU under optimal growth conditions. (**a**) Growth of strain GONU (blue) upon mineralization of DnOP (red). (**b**) Growth of strain GONU (blue) upon mineralization of DEHP (red). The status of the accumulation of metabolites is marked with different coloured lines. Vertical bars represent mean±sd values from triplicate measurements.

Strain GONU was able to utilize several LMW and HMW PAEs. Table 1 illustrates the utilization potential for six different PAEs, namely DMP, DEP, DBP, BBP, DnOP and DEHP, and the probable metabolic pathway intermediates of DnOP and DEHP. Utilization of DnOP and DEHP by *Gordonia* sp. strain GONU was validated by their removal from respective spent culture media with the concomitant increase in bacterial biomass (Fig. 2). In the process, DnOP was completely degraded by strain GONU within 20 h (extraction efficiency: 92.50±3.77 %), while its isomer DEHP (extraction efficiency: 91.83±2.28 %) was totally degraded within 24 h. Traces of both PA and PCA were identified as metabolites during DnOP utilization; however, MnOP, the other possible intermediate, could not be identified in HPLC analysis. On the other hand, PCA, PA and MEHP were detected during DEHP utilization.

**Table 1.** Substrate utilization profile for *Gordonia* sp. strain GONU*

Substrate	Growth rate (h^−1^)	Metabolic intermediate	Growth rate (h^−1^)
DMP	0.04	MnOP	nd
DEP	0.04	1-Octanol	0.04
DBP	0.08	1-Octanal	0.08
BBP	0.08	1-Octanoic acid	0.08
DnOP	0.28	MEHP	nd
DEHP	0.17	2-Ethyl-1-hexanol	0.04
Succinate	0.17	2-Ethyl-1-hexanal	0.08
Glucose	0.21	2-Ethyl-1-hexanoic acid	0.08
		PA/PCA	nd/0.04

*The concentration of each substrate and metabolic intermediate was 0.5 g l^–1^. nd, Not detected.

BBP, benzyl butyl phthalate; DBP, di-*n*-butyl phthalate; DEHP, di(2-ethylhexyl) phthalate; DEP, diethyl phthalate; DMP, dimethyl phthalate; DnOP, di-*n*-octyl phthalate; MEHP, mono(2-ethylhexyl) phthalate; MnOP, mono-*n*-octyl phthalate; PA, phthalic acid; PCA, protocatechuic acid.

### Identification of metabolic intermediates

**Fig. 3.**
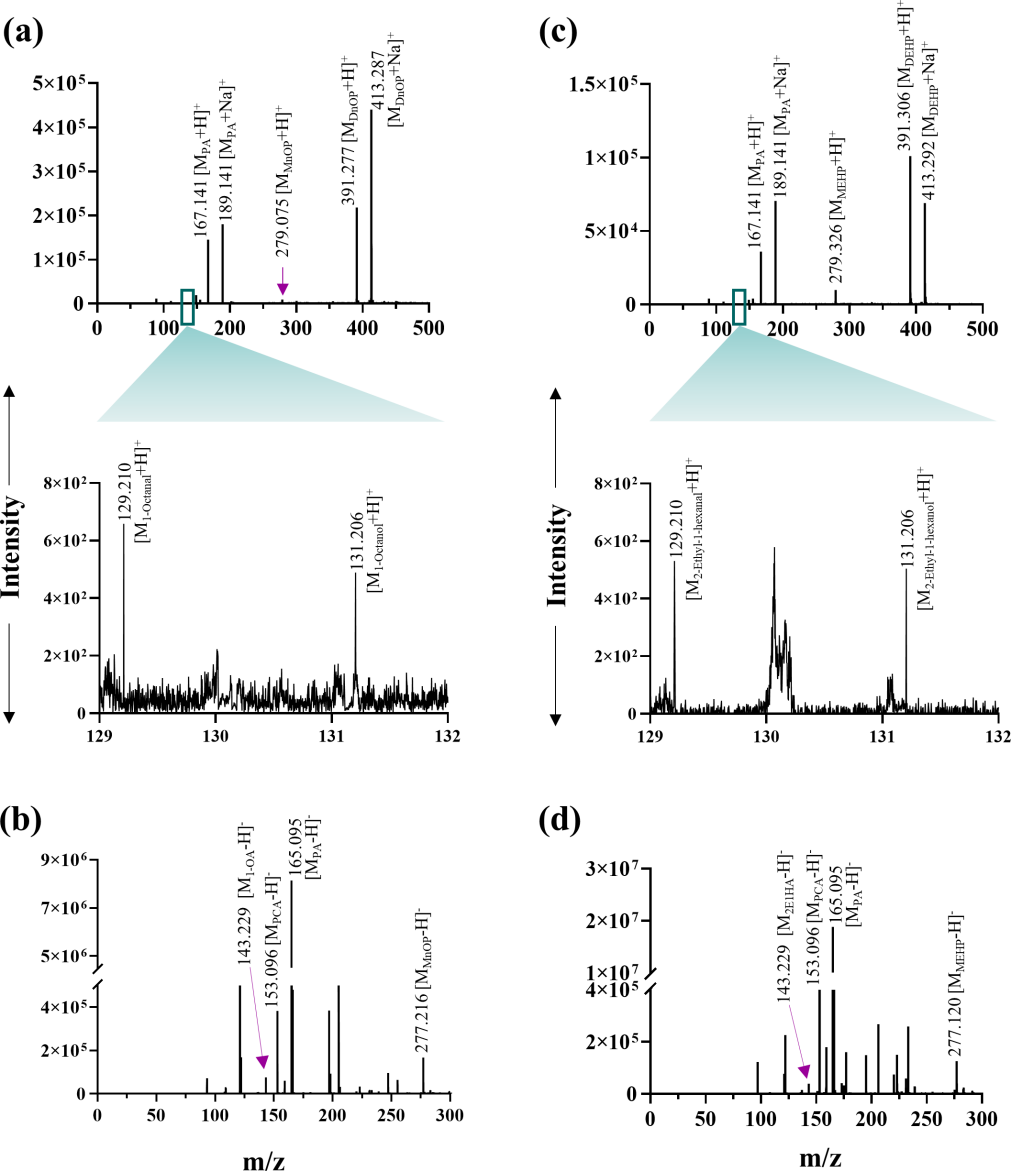
DI-ESI-HRMS analysis. Analysis of organic extracts of spent culture of strain GONU grown in the presence of DnOP in positive ion mode [M+H]^+^ or [M+Na]^+^ (**a**) and negative ion mode [M-H]^−^ (**b**) with the identification of MnOP, PA, PCA, 1-octanoic acid, and traces of 1-octanol and 1-octanal. Analysis of organic extracts of the spent culture of strain GONU, grown in the presence of DEHP in positive ion mode [M+H]^+^ or [M+Na]^+^ (**c**) and negative ion mode [M-H]^−^ (**d**) with the identification of MEHP, PA, PCA, 2-ethyl-1-hexanoic acid, and traces of 2-ethyl-1-hexanol and 2-ethyl-1-hexanal.

To identify metabolic intermediates, the ethyl acetate extract of resting cell cultures of DnOP-grown cells in the presence of DnOP was resolved by TLC (data not shown). Data revealed the identification of PA and PCA as metabolic intermediates based on comparion with authentic compounds. During similar research with DEHP, an additional metabolite was detected and identified as MEHP. To corroborate the metabolic profile, HPLC analysis was able to resolve the organic extract of DnOP-spent cultures of different growth phases, and three distinct peaks were observed (Fig. S3a). Based on the retention time, UV–visible spectra and co-elution profiles with reference standards, peaks I and II were identified as PCA (*R*_t_ 4.45 min) and PA (*R*_t_ 4.84 min), respectively, apart from the unutilized DnOP (peak III, *R*_t_ 43.18 min). Under identical analytical conditions, an organic extract of DEHP-spent culture revealed the presence of PCA (peak I, *R*_t_ 4.45 min), PA (peak II, *R*_t_ 4.84 min), MEHP (peak IV, *R*_t_ 35.44 min) and unconverted DEHP (peak V, *R*_t_ 43.43 min) (Fig. S3b). PCA was also identified as the metabolic intermediate in the resting cell incubation of PA with DnOP and DEHP-grown cultures. Further, DI-ESI-HRMS analysis identified all the metabolites obtained from the chromatographic analyses (TLC and HPLC), in addition to MnOP, 1-octanoic acid and 2-ethyl-1-hexanoic acid. In the DI-ESI-HRMS positive ion mode as protonated ions, [M+H]^+^, DnOP (*m/z* 391.277), and its four metabolites MnOP (*m/z* 279.075), PA (*m/z* 167.141), 1-octanol (*m/z* 131.206) and 1-octanal (*m/z* 129.210) were detected; whereas, in the case of DEHP (*m/z* 391.306), four metabolic intermediates, namely MEHP (*m/z* 278.332), PA (*m/z* 167.141), 2-ethyl-1-hexanol (*m/z* 131.206) and 2-ethyl-1-hexanal (*m/z* 129.210), were identified. In addition, DnOP (*m/z* 413.2873), DEHP (*m/z* 413.2915) and PA (*m/z* 189.141) were also identified as sodiated adducts, [M+Na]^+^. In the negative ion mode of DI-ESI-HRMS analysis, four metabolites of DnOP, namely MnOP (*m/z* 277.216), PA (*m/z* 165.095), PCA (*m/z* 153.096) and 1-octanoic acid (*m/z* 143.229), were detected. Analogously, PA, PCA, MEHP (*m/z* 276.303) and 2-ethyl-1-hexanoic acid (*m/z* 143.229) were identified in DEHP metabolism (Fig. 3). Thus, identification of metabolic intermediates revealed hydrolysis of DnOP and DEHP to PA via their respective monoesters followed by the metabolism of PA to PCA and that of the hydrolysed side chain alcohols (1-octanol and 2-ethyl-1-hexanol) to the corresponding acids via aldehydes, ultimately leading to TCA cycle intermediates.

### Oxygen uptake analysis

Molecular oxygen-dependent oxidation of the possible metabolic intermediates of DnOP and DEHP by cells grown individually on DnOP, DEHP or their possible metabolites as sole carbon and energy sources were determined polarographically (Table 2). DnOP-grown cells showed oxygen uptake in the presence of DnOP, MnOP, PA, PCA, 1-octanol and 1-octanal. In DEHP-grown cells, molecular oxygen consumption was noted in the presence of DEHP, MEHP, PA, PCA, 2-ethyl-1-hexanol and 2-ethyl-1-hexanal. However, PCA-grown cells of strain GONU showed oxygen uptake in the presence of PCA alone. Apart from cell respiration, no oxygen consumption was observed in succinate-grown cells, which was considered a negative control. Nevertheless, DnOP, DEHP and their corresponding monoesters are metabolized by hydrolytic enzyme(s), and oxygen consumption in the presence of these compounds can be explained due to molecular oxygen-dependent metabolism of their hydrolysed products (PA, 1-octanol and 2-ethyl-1-hexanol) and other lower pathway intermediates.

**Table 2.** Oxygen uptake rates with various compounds by resting cell transformation of *Gordonia* sp. strain GONU grown on different substrates*

Substrate	Oxygen uptake rate [(nmol O_2_ consumed) min^−1^ (mg of protein)^−1^] by cells grown on:			
	DnOP/DEHP	PCA	1-Octanoic acid/2-ethyl-1-hexanoic acid	Succinate
DnOP	35.76/nd	nd	nd/ nd	0.91
MnOP	7.56/nd	nd	nd/ nd	nd
PA	41.85/38.67	1.83	0.72/nd	1.88
PCA	63.41/74.51	78.01	nd/ nd	nd
1-Octanol	18.26/62.08	0.18	14.35/88.56	nd
1-Octanal	246.02/104.29	nd	227.94/114.74	nd
1-Octanoic acid	nd/ nd	nd	nd/ nd	nd
DEHP	nd/ 39.53	nd	nd/ 0.13	nd
MEHP	43.94/47.85	nd	nd/ nd	nd
2-Ethyl-1-hexanol	26.38/80.89	nd	17.97/93.17	nd
2-Ethyl-1-hexanal	338.05/143.97	nd	234.57/108.54	nd
2-Ethyl-1-hexanoic acid	nd/ nd	nd	nd/ nd	nd

*All values are corrected for endogenous O_2_ uptake. nd, Not detected.

### Enzyme assay

#### Spectrophotometric analysis of PCA

**Fig. 4.**
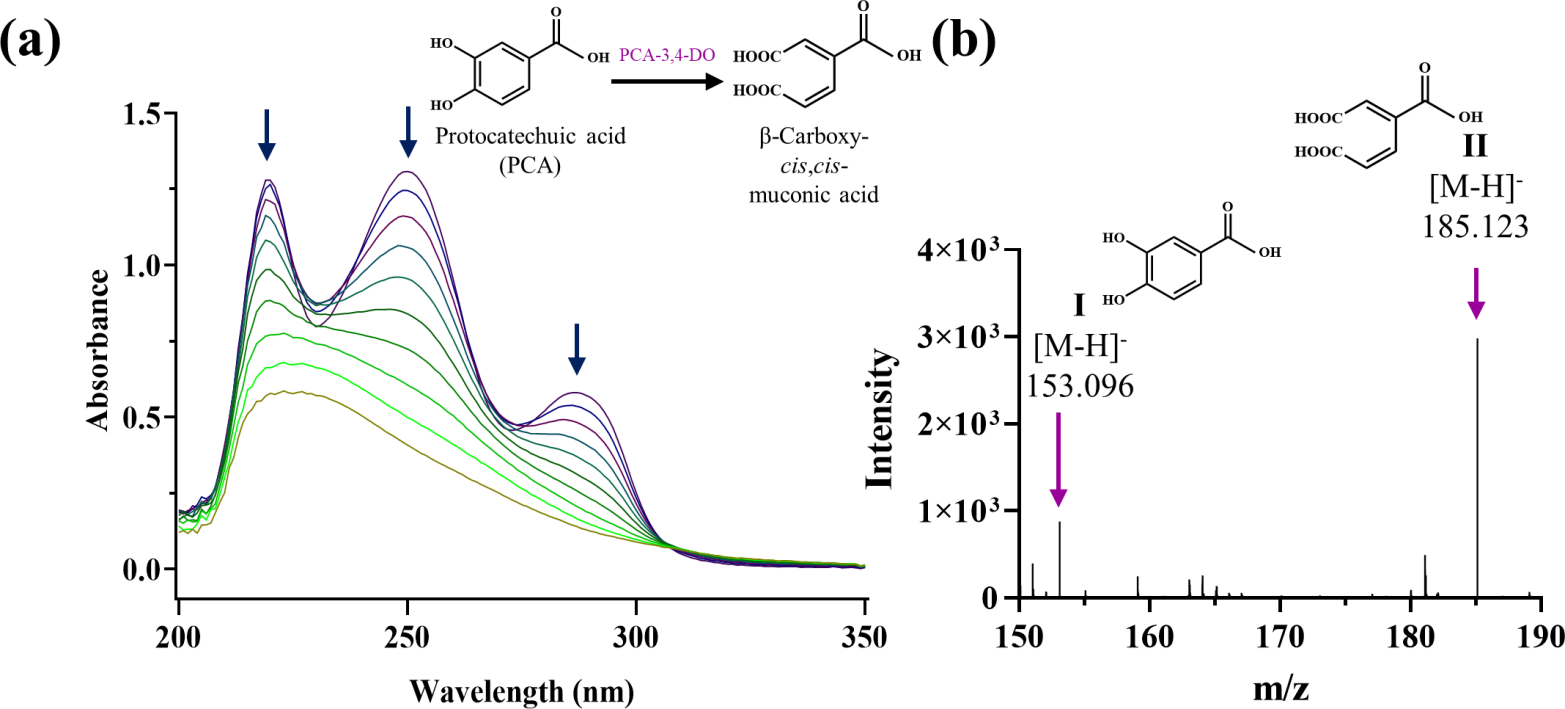
(a) Spectral changes during metabolism of PCA by the cell-free extract of DnOP-grown cells of *Gordonia* sp. strain GONU. The sample and reference cuvettes contained 50 mM potassium phosphate buffer (pH 7.0) in 1 ml volume. The sample cuvette contained 100 nmol PCA. Spectra were recorded at 1, 2, 3, 4, 5, 6, 7, 8, 9 and 10 min after the addition of 50 µg crude protein to both cuvettes. Down arrow indicates decreasing absorbance with time. (b) DI-ESI-MS analysis of cell-free extract-mediated transformation of PCA [peak I] to β-carboxy-*cis,cis*-muconic acid [peak II] in DnOP-grown cells of strain GONU, analysed in negative ion mode [M-H]^−^. The reaction was conducted at 28 °C for 5 min using 100 µg of cell-free extract. Identification of β-carboxy *cis,cis*-muconic acid (II) by DI-ESI-HRMS analysis of the organic extract of the reaction mixture containing the cell-free extract (100 µg of protein) of DnOP-grown cells of strain GONU and protocatechuic acid (PCA, I), incubated for 10 min.

For the identification of the *ortho-* or *meta*-cleavage pathway of metabolism of PCA, crude CFEs of strain GONU grown in the presence of DnOP and DEHP were incubated separately with PCA to monitor a possible change in colour of the reaction mixtures. Following incubation, no colour was developed, eliminating the possibility of forming a *meta*-cleavage product (2-hydroxy-4-carboxymuconic semialdehyde, yellow in colour) from PCA [36] and indicated a possible involvement of *ortho*-cleavage dioxygenase in PCA metabolism. To substantiate this, spectrophotometric analysis of the conversion of PCA to β-carboxy-*cis,cis*-muconate by the CFE of strain GONU, grown on DnOP, revealed a decrease in absorbance at 254 and 290 nm (Fig. 4a), a characteristic feature of the involvement of *ortho*-cleavage dioxygenase [37–39]. Similar spectral changes were observed with the CFEs of strain GONU, grown on DEHP and PCA. No such CFE-mediated ring cleavage was found in succinate-grown cells. Nevertheless, a trace amount of β-carboxy-*cis,cis*-muconate was detected in the DI-ESI-HRMS analysis of the organic extract of the cell-free extract-mediated PCA-transforming reaction mixture, supporting the spectral changes in PCA metabolism (Fig. 4b). Based on the ring cleavage activity, it appeared that either PCA or one of its metabolites is the inducer of the PCA ring cleavage enzyme.

#### NAD(P)^+^-independent dehydrogenase assay and β-oxidation pathway

A DCPIP reduction assay was performed using the crude CFEs of the test organism grown in the presence of DnOP or DEHP to substantiate the oxygen uptake profile in the metabolism of side-chain alcohols and their dehydrogenated counterparts (Table 2), as well as the possible involvement of NAD(P)^+^-independent alcohol and aldehyde dehydrogenases [31]. Following incubation at 28 °C, a change in colour of the reaction mixtures with respect to the control was observed (Fig. S4). DnOP- and 1-octanoic acid-grown cultures exhibited NAD(P)^+^-independent alcohol and aldehyde dehydrogenase activities toward both 1-octanol and 1-octanal. Similarly, a visible change in colour was observed with CFE obtained from DEHP- and 2-ethyl-1-hexanoic acid-grown cultures when 2-ethyl-1-hexanol and 2-ethyl-1-hexanal were used individually as assay substrates. In either case, no DCPIP reduction was observed with CFE obtained from succinate-grown culture. Thus, in the DnOP and DEHP degradation pathways, the side-chain alcohols were metabolized to their dehydrogenated products, aldehydes and acids by oxygen-consuming NAD(P)^+^-independent dehydrogenase(s) and the corresponding gene(s) is considered to be upregulated by the alkanoic acids.

The metabolites, 1-octanoic acid from 1-octanol and 2-ethyl-1-hexanoic acid from 2-ethyl-1-hexanol, were further metabolized via the β-oxidation pathway, and this was confirmed using acrylic acid, a β-oxidation pathway inhibitor that inhibits 3-ketoacyl-CoA thiolase activity, the final step of the pathway. When acrylic acid (5 mM) was mixed with 1-octanoic acid or 2-ethyl-1-hexanoic acid in MSM, no growth of strain GONU was observed, in contrast to the control experiments (which showed positive growth) with succinate and PCA as substrates (PCA is processed via the β-ketoadipate pathway) under similar growth conditions (Fig. S5).

### A synopsis of genome sequencing and analysis of PAE catabolic genes

The *de novo* whole genome sequence of *Gordonia* sp. strain GONU revealed the presence of a single chromosome, from which 5 583 881 bp was identified with a GC content of 67.3 %. A total of 5041 protein-coding genes were annotated, and the genome annotation revealed 100 % genome completeness. Overall, one operon for rRNA and 48 operons for tRNA were annotated in the genome. Additionally, 44 genes were predicted to be involved in the metabolism of aromatic compounds according to the COG proteins database (Fig. S6). Average nucleotide identity (ANI) analysis of the genome sequence of strain GONU resulted in 98.13 % identity with *Gordonia amicalis* CCMA-559 (GenBank accession number NZ_AWTB00000000.1). In the genome of strain GONU, an aggregate of 178 hydrolases, 55 esterases, 21 dioxygenases, 16 decarboxylases, 189 dehydrogenases and a large number of oxido-reductases were annotated. Based on the presence of a large number of esterases that can hydrolyse a wide range of substrates [40], strain GONU is considered to possess substantial hydrolytic potential for the degradation of various xeno-oestrogens.

### Proteome analysis

**Fig. 5.**
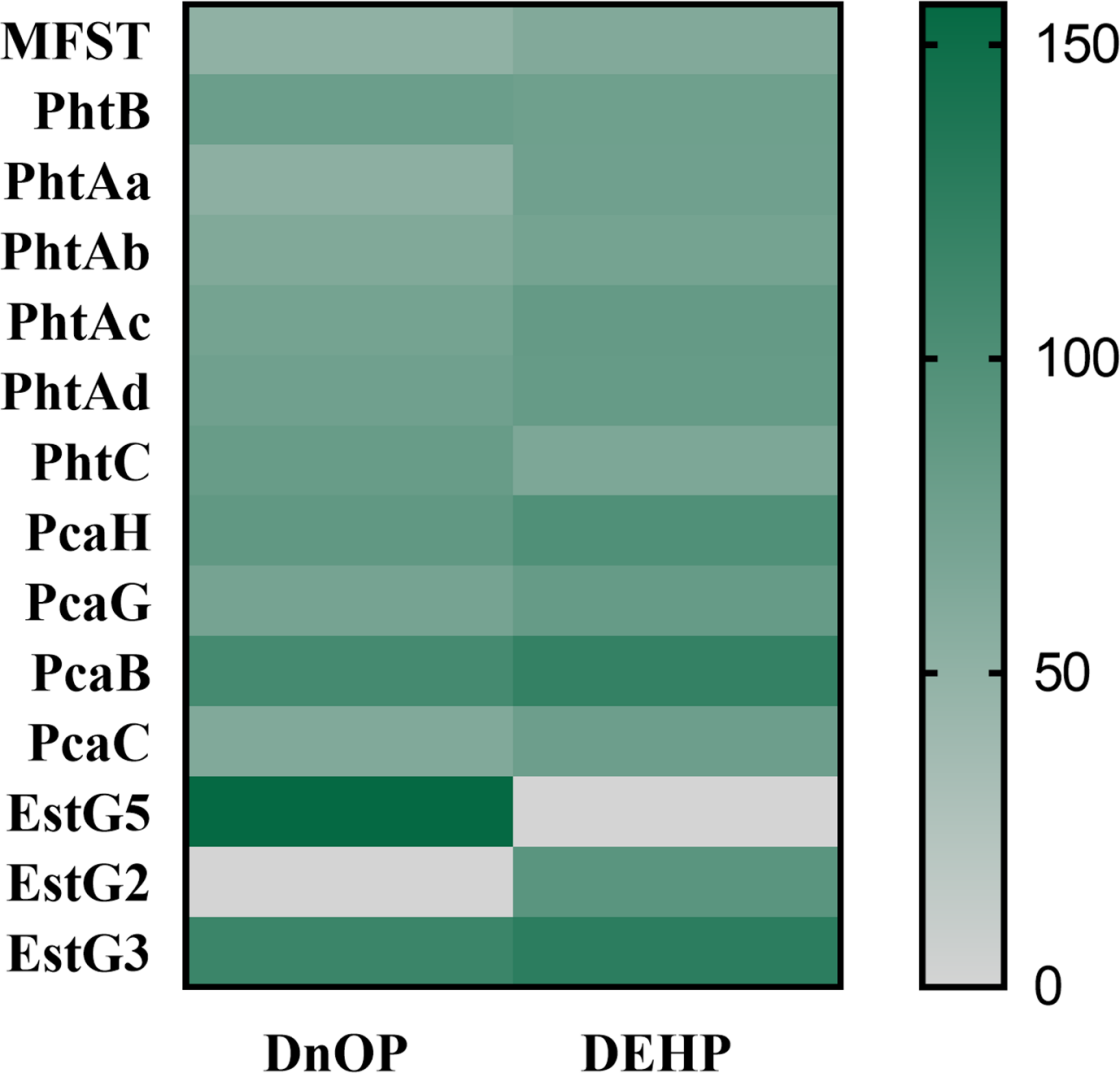
Heatmap profile of differentially expressed proteins in DnOP- and DEHP-grown cells of strain GONU. Equal amounts of cell-free extract from the DnOP-, DEHP- and succinate-grown cells were subjected to ESI-LC-MS/MS. The abundance of PAE-degrading pathway-specific proteins was normalized with respect to that obtained from the succinate-grown cells, used as a control. Relative abundances are represented by colour (grey to green from lower to higher abundance), as indicated in the vertical legend bar.

**Fig. 6.**
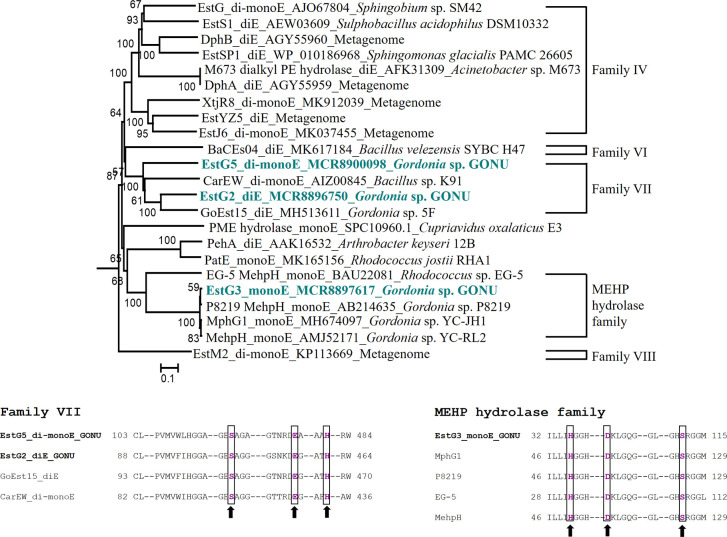
Phylogenetic relationship of upregulated phthalate hydrolases of strain GONU. The phthalate hydrolases (EstG2, EstG3 and EstG5) are shown in bold type along with their functional type (diE, monoE and di-monoE) and family classification. Numbers at nodes indicate the levels of bootstrap support based on neighbour-joining analysis of 100 resampled data sets. Bootstrap values below 50 % are not shown. Bar, 0.1 substitutions per amino acid position. The GenBank accession numbers of the sequences are shown prior to the name of organisms/metagenome. Multiple sequence alignment was performed using ClustalX2 and the phylogenetic tree was reconstructed using the neighbour-joining algorithm as implemented in Tree Explorer 2.12. diE, monoE and di-monoE represent diesterase, monoesterase and diesterase-monoesterase, respectively. Multiple sequence alignments of EstG5 and EstG2 afﬁliated to family VII, and of EstG3 affiliated to the MEHP hydrolase family, along with the representative enzyme sequences of respective families showing conserved sequence motifs. Amino acid residues responsible for the formation of the catalytic triad are written in bold type and marked in black boxes and shown with arrows. Alignment was performed with default parameters using ClustalX2.

The peptide profiles in the trypsin-digested cell lysates of DnOP-, DEHP- and succinate-grown cultures showed differential expression of several proteins analysed by LC-ESI-MS/MS (Fig. 5). The six enzymes responsible for the metabolism of PA to PCA and the four enzymes responsible for the metabolism of PCA to β-ketoadipate enol-lactone via β-carboxy-*cis,cis*-muconic acid showed upregulation in DnOP- and DEHP-grown cultures of strain GONU compared to succinate-grown culture. In parallel, among the 233 annotated esterases and hydrolases present in the genome of strain GONU, only three esterases/hydrolases were differentially expressed (>90-fold) in both DnOP- and DEHP-grown cultures in comparison to succinate-grown culture. One of the overexpressed enzymes, EstG3, was found to be common in both DnOP- and DEHP-grown cultures (fold change: 120.864 in DnOP; 126.155 in DEHP). By contrast, comparing the hydrolase intensities with respect to succinate-grown culture, EstG5 (fold change: 156.346) was overexpressed only in the DnOP-grown culture but not in the DEHP-grown culture. On the other hand, EstG2 (fold change: 91.457) was overexpressed in the DEHP-grown culture but not in the DnOP-grown culture. In addition to the above, relatively low overexpression of EstG1 (fold change: 9.829 in DnOP; 9.451 in DEHP) and EstG4 (fold change: 3.248 in DnOP; 4.794 in DEHP) was observed with respect to the succinate-grown cultures. However, neither EstG1 nor EstG4, showed differential mRNA expression in RT-PCR analysis; rather, these two relatively abundant esterases/hydrolases appeared constitutive in nature (Fig. S7). Further, to evaluate the nature of these induced hydrolases/esterases, EstG2, EstG3 and EstG5 sequences were compared with the previously reported PAE hydrolases following the modified esterase classification [41], and it appeared that EstG2 and EstG5 belong to family VII (phthalate diesterase and diesterase-monoesterase), whereas EstG3 belongs to the MEHP hydrolase (phthalate monoesterase) family (Fig. 6).

### Transcriptional analysis

**Fig. 7.**
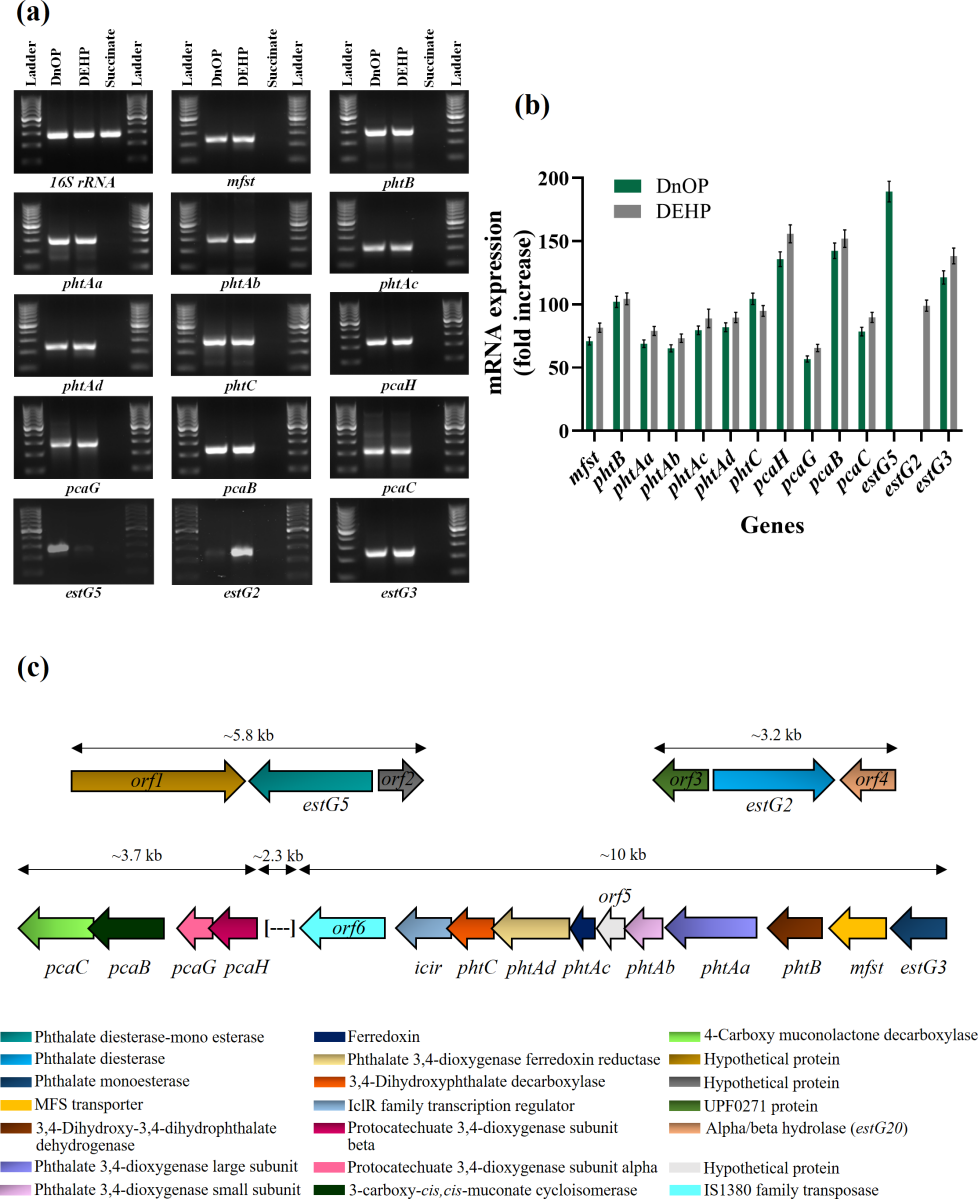
(**a**) Agarose gel electropherogram profile showing RT-PCR analysis of mRNA transcripts of pathway genes involved in the catabolism of DnOP and DEHP from DnOP-, DEHP- and succinate-grown cells of strain GONU. A ladder (100 bp) was used as a marker. (**b**) Bar graph representing fold change of the transcripts of the catabolic genes, quantified by RT-qPCR. Succinate-grown cells were taken as the negative control, while 16S rRNA was kept as the endogenous control. Average values were obtained from triplicate experiments. The data presented are mean±sd. (**c**) Genetic organization of *estG5* (locus tag NWF34_24470), *estG2* (locus tag NWF34_07225), *estG3-pht* (locus tag NWF34_11730 to NWF34_11690) and *pca* (locus tag NWF34_11665 to NWF34_11650) gene clusters in *Gordonia* sp. strain GONU along with the gene designations.

Differential gene expression by RT-PCR analysis was performed to validate the differential proteome profile and the inducible nature of esterases, and that of *pht* and *pca* gene clusters, and the results are shown in Fig. 7(a, b). Data revealed that all six phthalic acid-metabolizing genes (*phtBAaAbAcAdC*) of the *pht* gene cluster, four genes of the PCA-degrading gene cluster (*pcaHGBC*), one transporter gene (*mfst*) and three esterases (*estG2*, *estG3* and *estG5*) showed differential mRNA expression in either DnOP- or DEHP-grown cultures with respect to succinate-grown cultures of strain GONU. The organization of all the upregulated genes/operons is depicted in Fig. 7(c). With respect to the genome sequence, the locus for PhtCAdAcOrf5PhtAbAaBMFSTEstG3 is MCR8897609–MCR8897617, the locus for PcaCBGH is MCR8897601–MCR8897604, and those for EstG5 and EstG2 are MCR8900098 and MCR8896750, respectively (Fig. 7c). The only difference between the DnOP- and DEHP-grown cultures is the differential mRNA expression of *estG5* in the former and that of *estG2* in the latter. However, no such amplified products were observed when MnOP-succinate, MEHP-succinate, PA-succinate or succinate was used as a growth substrate (Fig. S8). Further, end-point PCR experiments revealed that the inducible *estG3* was co-transcribed with *mfst* and *pht* genes, indicating the genes present in the locus MCR8897609–MCR8897617 are under the control of a specific regulation (Fig. S9). Similarly, *estG2*, *estG5* and PCA-metabolizing *pca* gene cluster are considered to be upregulated by specific inducers.

### Mutational study

**Fig. 8.**
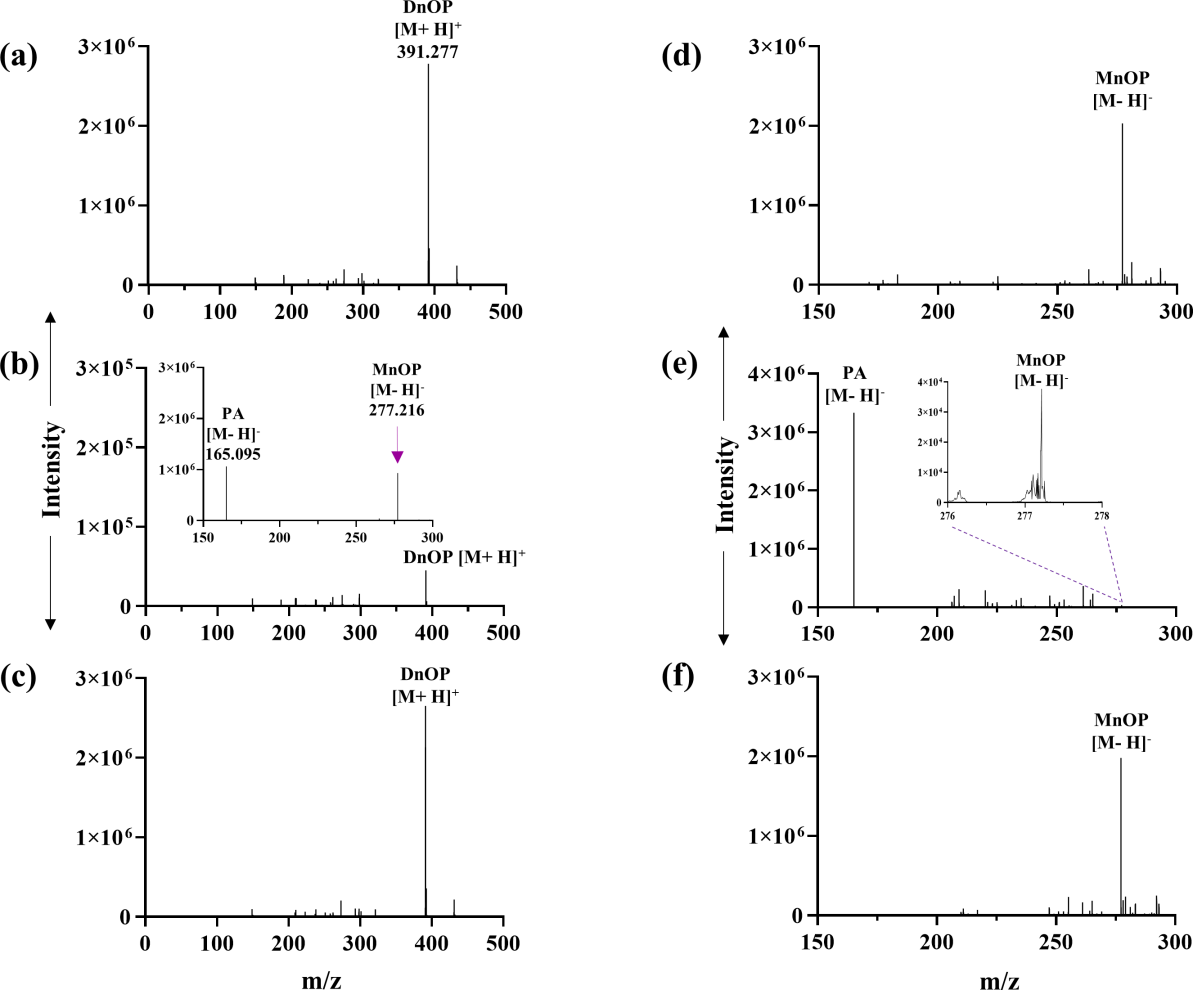
DI-ESI-HRMS analysis of the organic extracts of the reaction mixtures containing DnOP-induced cell-free extracts (CFEs) of wild-type and mutant (*ΔestG5*) strains and DnOP or MnOP, as substrate. Authentic DnOP and MnOP, used as substrate controls, were analysed in positive ion mode [M+H]^+^ (**a**) and negative ion mode [M-H]^−^ (**d**), respectively. The metabolic profiles of DnOP with the CFE obtained from DnOP-induced wild-type strain (**b**) and mutant strain (**c**) were analysed in positive ion mode [M+H]^+^. Metabolic profiles of MnOP with the CFE obtained from DnOP-induced wild-type strain (**e**) and mutant strain (**f**) were analysed in negative ion mode [M-H]^−^. The inset shown in (**b**) was analysed in negative ion mode [M-H]^−^ while the inset shown in (**e**) is the zoom-in profile to detect residual MnOP. For each reaction, 0.5 g l^−1^ of the substrate and 100 µg of CFE were used and incubated for 1 h.

**Fig. 9.**
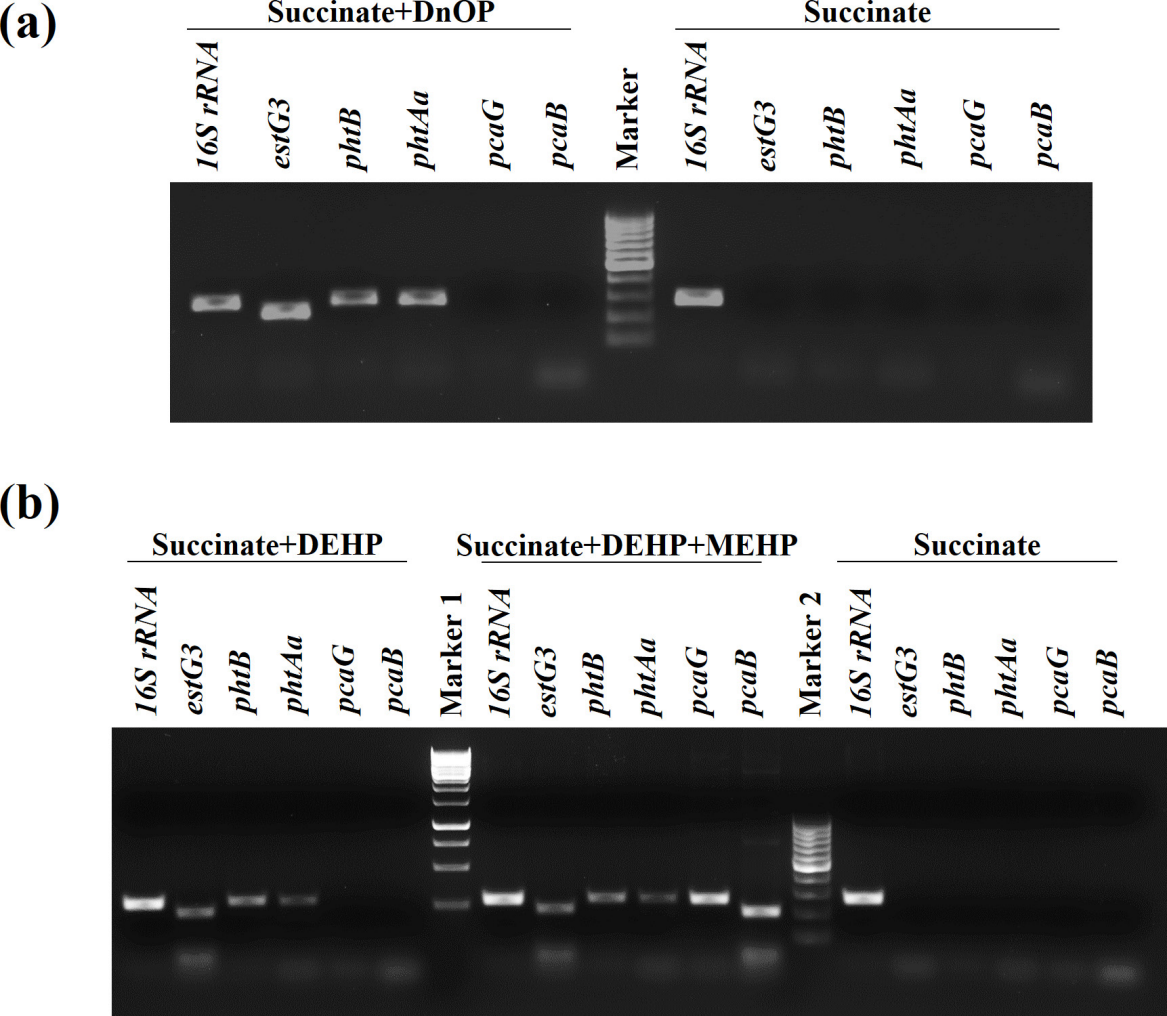
Agarose gel electropherogram profile showing RT-PCR analysis of mRNA transcripts of *estG3* and *pht* genes in *ΔestG5* (**a**) and *ΔestG2* (**b**) mutants of strain GONU. The DnOP+succinate, DEHP+succinate and DEHP+MEHP+succinate-induced cells were taken as test samples, while the succinate-grown cells were taken as a negative control. The 16S rRNA gene was kept as an endogenous control.

To evaluate the functional nature of EstG5 and EstG2, and the role of DnOP and DEHP, respectively, in the inducible regulation of metabolic pathways, *estG5* and *estG2* gene disruptions were corroborated independently by the insertion of a *kan* cassette in the chromosome of the wild-type strain. The resultant *ΔestG5* mutant strain could not utilize DnOP, but growth was observed in the presence of DEHP, PCA, DnOP-PA, DnOP-MEHP, the hydrolysed alcohols and their dehydrogenated products in MSM (Fig. S10a). However, no growth was observed in MSM-DnOP-MnOP medium. In a respirometric assay with the DnOP-induced *ΔestG5* mutant, no molecular oxygen consumption was detected in the presence of DnOP, MnOP, 1-octanol, 1-octanal or PCA as an assay substrate, but oxygen uptake was observed with PA. The mutant strain’s inability to metabolize 1-octanol or 1-octanal in DnOP-induced cell-free extract was also reflected in the DCPIP assay (Fig. S4). However, the *ΔestG5* mutant grown in the presence of DEHP was able to metabolize 2-ethyl-1-hexanol, 2-ethyl-1-hexanal, 1-octanol and 1-octanal, indicating wide substrate specificity of the inducible NAD(P)^+^-independent dehydrogenase(s), and the mutant has no adverse effect on the complete metabolism of DEHP (Fig. S10a). Additionally, in the CFE-mediated experiment, the DnOP-induced *ΔestG5* mutant failed to convert DnOP and MnOP, unlike the wild-type strain, as analysed by DI-ESI-HRMS (Fig. 8). Further, in RT-PCR analysis, DnOP-induced mutant culture showed upregulation of all the components of *the estG3-pht* gene cluster but failed to induce the *pca* gene cluster (Fig. 9a). In all the experiments, kan50 was used as a selection marker, and 0.5 g l^−1^ DnOP was used as an inducer. Thus, the *ΔestG5* mutant cannot utilize DnOP due to the knockout of DnOP diesterase and MnOP monoesterase (a diesterase-monoesterase, EstG5) but can induce the *estG3-pht* gene cluster in the presence of DnOP, and has no adverse impact on the DEHP metabolic pathway.

**Fig. 10.**
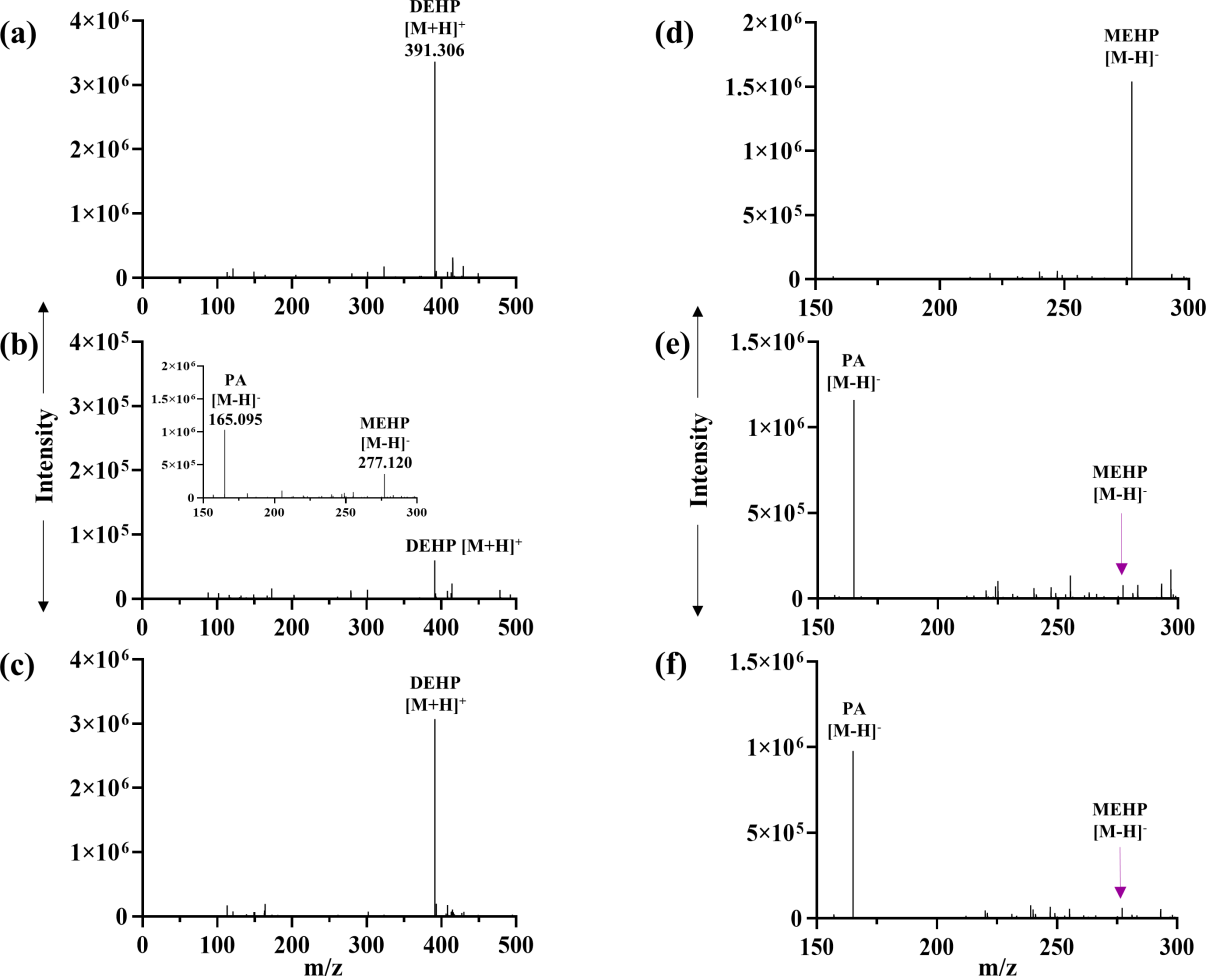
DI-ESI-HRMS analysis of the organic extracts of the reaction mixtures containing DEHP-induced cell-free extracts (CFEs) of wild-type and mutant (*ΔestG2*) strains with DEHP or MEHP as substrate. Authentic DEHP and MEHP, used as substrate controls, were analysed in positive ion mode [M+H]^+^ (**a**) and negative ion mode [M-H]^−^ (**d**), respectively. The metabolic profiles of DEHP with the CFE obtained from DEHPP-induced wild-type strain (**b**) and mutant strain (**c**) were analysed in positive ion mode [M+H]^+^. Metabolic profiles of MEHP with the CFE obtained from DEHP-induced wild-type strain (**e**) and mutant strain (**f**) were analysed in negative ion mode [M-H]^−^. The inset shown in (**b**) was analysed in negative ion mode [M-H]^−^ while the inset shown in (**e**) is the zoom-in profile to detect residual MEHP. For each reaction, 0.5 g l^−1^ of the substrate and 100 µg of CFE were used and incubated for 1 h.

In the *ΔestG2* mutant, the recombinant strain could not utilize DEHP as a sole carbon source for its growth, but growth was observed in DnOP, PCA, DEHP-MEHP, DEHP-PA, the hydrolysed alcohols and their dehydrogenated products in MSM (Fig. S10b). In respirometric analysis with the DEHP-induced *ΔestG2* mutant, molecular oxygen consumption could not be detected in DEHP, PCA, 2-ethyl-1-hexanol and 2-ethyl-1-hexanal but O_2_ uptake was observed when MEHP and PA were used as assay substrates (Table S3). In a CFE-mediated transformation assay using DEHP-grown wild-type cells, DEHP and MEHP were metabolized to PA. Unlike the wild-type strain, the CFE of the DEHP-induced *ΔestG2* cells failed to convert DEHP, but MEHP could be converted to PA (Fig. 10). Additionally, the *estG3-pht* gene cluster was upregulated in RT-PCR analysis with DEHP-induced *ΔestG2* mutant cells. In contrast, the DEHP-MEHP-grown *ΔestG2* mutant culture showed upregulation of *estG3*, *pht* and *pca* genes (Fig. 9b). The possibility of catabolic repression due to the presence of succinate could be eliminated as the *estG3-pht* operon showed upregulation in *ΔestG5* and *ΔestG2* mutant cells grown in MSM in the presence of succinate-DnOP and succinate-DEHP, respectively, using 2 mM succinate. In all the experiments, kan50 was used as the selection marker, and 0.5 g l^−1^ DnOP or DEHP was used as an inducer molecule. Thus, the *ΔestG2* mutant cannot utilize DEHP due to the knockout of DEHP diesterase but can induce the *estG3-pht* gene cluster in the presence of DEHP, and has no adverse impact on the DnOP metabolic pathway.

In cells of both mutants, as the *estG3-pht* operon is induced in the presence of DnOP or DEHP, oxygen uptake was observed for PA (but not for MnOP as EstG3 cannot metabolize MnOP) in the *ΔestG5* mutant and that for MEHP and PA in the *ΔestG2* mutant, as for wild-type cells (Table S3). On the other hand, the genes responsible for the metabolism of PCA and side-chain alcohols or their metabolites remain uninduced in mutant cells, and as such the oxygen uptake rates were almost negligible for all these lower metabolites in comparison to wild-type cells. Additionally, to verify the role of EstG5 and EstG2, the *ΔestG5* and *ΔestG2* mutants were respectively incubated in the presence of DnOP and DEHP supplemented with the corresponding cell-free extract of DnOP- and DEHP-grown cells of the wild-type strain, which resulted in growth of the mutant strains (Fig. S11).

## Discussion

Recently, the genus *Gordonia* has attracted growing environmental and biotechnological attention owing to the potential of its members to degrade, transform and synthesize a large number of aromatics, including oestrogenic PAEs [41]. Hitherto, 18 PAE-degrading strains belonging to the genus *Gordonia* were identified from various environmental sources. Among them, 14 strains could individually utilize PAEs (mostly LMW PAEs). On the other hand, other strains were involved as a component of co-culture in the degradation of PAEs, predominantly elucidating biodegradation potential with a few illustrating metabolic pathways [6, 42 and references therein]. To date, the whole genome sequences for six different PAE-degrading *Gordonia* species have been reported, but only a few reports are available on the characterization of catabolic pathways of degradation of PAEs at the gene level [43–47].

**Fig. 11.**
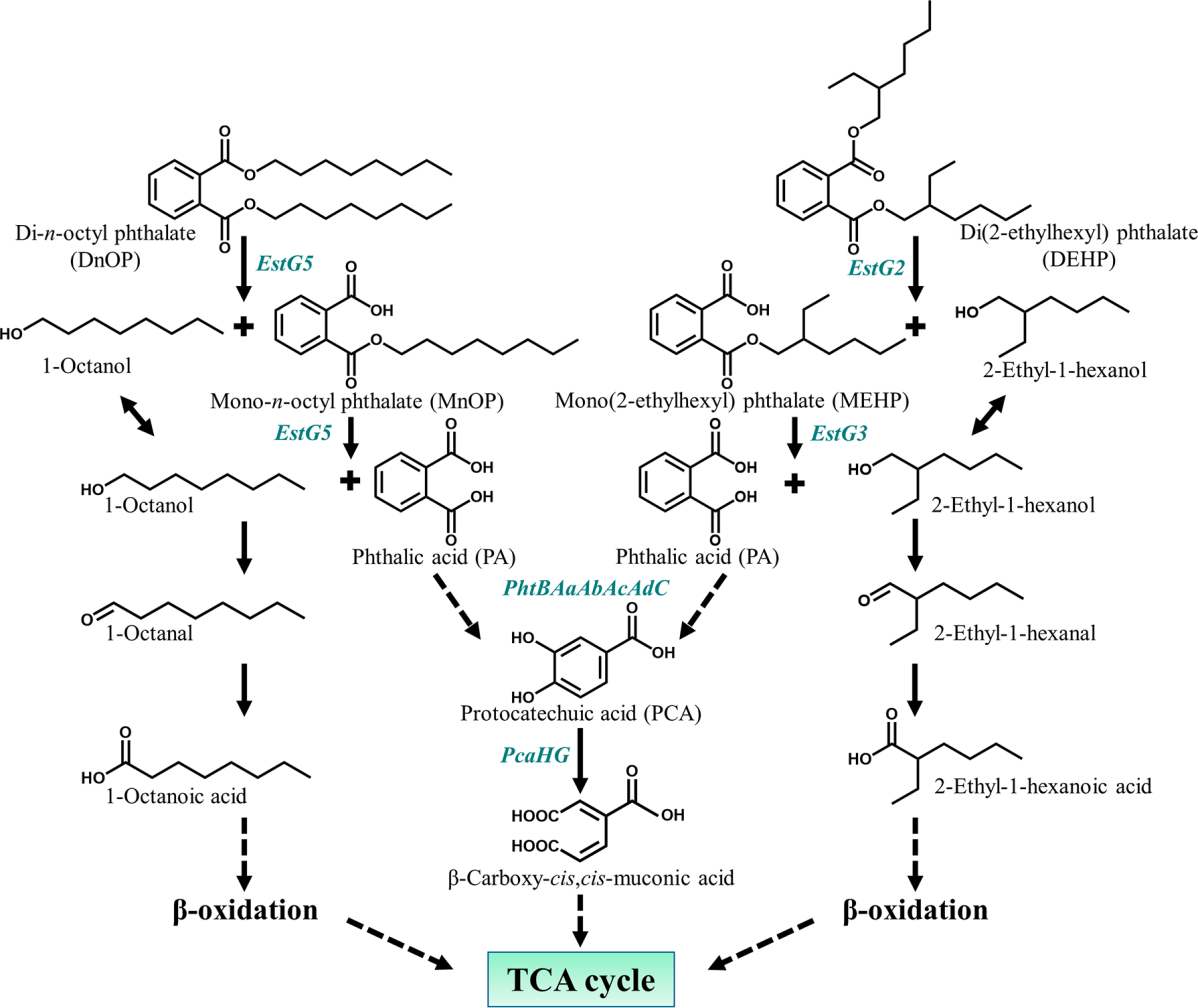
Metabolic pathways for the complete assimilation of DOP isomers (DnOP and DEHP) in *Gordonia* sp. strain GONU.

The present communication reports the most comprehensive multi-omics study evaluating the regulation of the inducible hydrolytic pathways of the metabolism of DnOP and DEHP in a *Gordonia* species at the molecular level. In the hydrolytic pathway, the involvement of a diesterase-monoesterase (EstG5) in the degradation of DnOP and a combination of diesterase (EstG2) and monoesterase (EstG3) for that of DEHP were evaluated. Further, in the metabolism of one of the hydrolysed products, PA, the classical phthalate and protocatechuate metabolic pathways involving phthalate 3,4-dioxygenase and protocatechuate 3,4-dioxygenase, respectively, were ascertained. For the hydrolysed side-chains, both 1-octanol (from DnOP) and 2-ethyl-1-hexanol (from DEHP) and their dehydrogenated counterparts showed oxygen-dependent metabolism (Fig. S4 and Table 2), unlike available reports on the metabolism of PAE-hydrolysed side-chain alcohols, where typically oxygen-independent alcohol and aldehyde dehydrogenases [NAD(P)^+^ as a cofactor] are involved [48]. Thus, MnOP, PA, PCA, β-carboxy-*cis,cis*-muconate, 1-octanol, 1-octanal and 1-octanoic acid were identified as metabolic intermediates in the degradation of DnOP, while MEHP, PA, PCA, β-carboxy-*cis,ci*s-muconate, 2-ethyl-1-hexanol, 2-ethyl-1-hexanal and 2-ethyl-1-hexanoic acid were identified for DEHP, as depicted in the proposed metabolic pathways (Fig. 11).

Both DnOP and DEHP degradation pathways in strain GONU are inducible, and the pathways comprise multiple inducible degradative operons (Fig. 7c). The initial enzymes responsible for hydrolysing DnOP and DEHP, identified as EstG5 and EstG2, are induced only by their respective substrates. The next catabolic operon encoding EstG3, a monoesterase, and PhtBAaAbAcAdC, comprising multicomponent phthalate 3,4-dioxygenase, a dihydrodiol dehydrogenase and a decarboxylase, involved in the transformation of PA to PCA, could be induced by both DnOP and DEHP. In addition to the above, the genome harbours a *pca* operon, encoding PcaHGBC, consisting of a two-component protocatechuate 3,4-dioxygenase, a carboxy-*cis*,*cis*-muconate cycloisomerase and a carboxymuconolactone decarboxylase, which can convert PCA to β-ketoadipate enol-lactone, was induced by protocatechuic acid, a common intermediate of DnOP and DEHP metabolism or its metabolite. The side-chain alcohols, 1-octanol and 2-ethyl-1-hexanol, the respective hydrolysed products of DnOP and DEHP, were metabolized by inducible NADP^+^-independent dehydrogenase(s) induced probably by the side-chain derived alkanoic acids (Table 2, Fig. S4).

Interestingly, EstG5 appeared as a diesterase-monoesterase capable of transforming DnOP to PA via MnOP. On the other hand, EstG3, a monoesterase induced in the presence of both DnOP and DEHP, could not convert MnOP. Phylogenetic analysis of the three identified esterases, in combination with the reported phthalate esterases and hydrolases, showed that EstG2 and EstG5 belong to the family VII esterases and shared all nine conserved domains and motifs, including the catalytically essential pentapeptide motif GXSAG, reported for phthalate esterases of this particular family. EstG5 appeared phylogenetically closest to CarEW from *Bacillus* sp. K91, the only diesterase-monoesterase belonging to family VII reported so far [20], showing 36.6 % identity, whereas EstG2, the diesterase belonging to this family, showed a maximum identity of 58.24 % with GoEst15 from *Gordonia* sp. 5F [49]. EstG3, the monoesterase, on the other hand, exhibited 99.66 % similarity to MehpH from *Gordonia* sp. P8219 [50], which belongs to the MEHP hydrolase family and shares seven conserved motifs, including motifs harbouring the catalytic triad residues Asp-Ser-His (Fig. 6) [51].

The other interesting feature is the inability of strain GONU to utilize PA, despite its genome encoding all the essential genes for the metabolism of PA. Nevertheless, analyses revealed that the PA degradative operon is fused with EstG3 (a monoesterase belonging to the MEHP family of esterases). The fused operon, *estG3-pht*, is induced in the presence of DnOP and DEHP but not in the presence of MnOP, MEHP or PA. Nevertheless, strain GONU could not utilize MnOP or MEHP due to the associated inducible features of relevant catabolic genes.

The distinct molecular architecture of coordinated regulation in the metabolism of DnOP and DEHP, as revealed by the biochemical, proteomic and RT-PCR analyses in the wild-type strain, was further verified using *the ΔestG5* and *ΔestG2* mutants. Studies showed that the *ΔestG5* mutant could not utilize DnOP but remained capable of utilizing DEHP, PCA and the side-chain alcohols (1-octanol and 2-ethyl-1-hexanol) as sole carbon sources. Growth of the mutant in MSM supplemented with DnOP+PA confirmed DnOP’s role as an inducer of the *estG3-pht* fused operon (Fig. S10a). Again, the inability of the *ΔestG5* mutant to grow in the presence of DnOP+MnOP and the inability of the CFE of the mutant, grown in the presence of DnOP+succinate, to convert DnOP or MnOP indicate that EstG5 possesses both DnOP diesterase and MnOP monoesterase activities (Fig. 8). Again, EstG3-mediated MnOP monoesterase activity could not be revealed in the mutant strain grown in the presence of DnOP+succinate, even though the fused operon *estG3-pht* is induced by DnOP (Figs 8 and 9a). On the other hand, the mutant strain can grow in the presence of DEHP and can also induce the *estG3-pht* operon, with DEHP-inducible EstG2, a diesterase that can convert DEHP into MEHP, and EstG3, a monoesterase that can convert MEHP into PA (Figs 9b and 10). Additionally, the CFEs prepared from the DnOP- or DEHP-grown culture of the wild-type strain and those obtained from the DnOP-succinate-grown culture of the *ΔestG5* mutant strain are capable of transforming MEHP to PA, which implies that the DnOP/DEHP-inducible EstG3 monoesterase is functionally specific towards MEHP but not MnOP (Figs S12, 8f and 10e).

On the other hand, the results of substrate utilization, induction profiling by RT-PCR and CFE-mediated substrate transformation assays using the *ΔestG2* mutant further substantiated the nature of EstG2 and its role in DEHP metabolism. Expectedly, the mutant strain failed to utilize DEHP as a sole carbon source. However, growth was seen in MSM supplemented with DEHP-MEHP and DEHP-PA, again supporting the role of DEHP as the inducer of the *estG3-pht* operon, involved in MEHP and PA metabolism. Monoesterase activity of the *estG3* gene product towards MEHP in the *ΔestG2* mutant was validated both under *in vivo* and *in vitro* conditions (Figs 10f and S10b). Again, the upregulation of *pca* genes in DEHP-MEHP-grown *ΔestG2* mutant cells indicated the metabolism of PA to PCA by the DEHP-inducible *estG3-pht* operon (Fig. 9b). Thus, EstG2 in strain GONU is a diesterase and is specific towards DEHP as a substrate.

To date, very little is known about the regulation of genes and operons involved in the degradation of PAEs, although the whole genome sequences harbour a plethora of esterases and hydrolases and a very large number of annotated catabolic genes of relevance. However, in a recent study, a glimpse of inducible regulation of the degradation of PAEs was reported based on proteogenomic and metabolomic approaches, but the study failed to confirm the initial enzyme(s) involved in the metabolic process [52]. To the best of our knowledge, the present study has deciphered a distinctive molecular architecture and an atypical inducible regulation of genes and operons delineating three different esterases and other essential catabolic genes in the complete degradation of oestrogenic DOP isomers that are largely used as plasticizers.

In summary, the bacterial strain GONU, belonging to the genus *Gordonia *, isolated from a municipal waste-contaminated soil sample, was capable of utilizing an array of phthalate diesters, starting from methyl to octyl side chains. A comprehensive biochemical and molecular analysis revealed that strain GONU is able to hydrolyse DnOP and its isomer DEHP by using a diesterase-monoesterase (EstG5) and a combination of a diesterase (EstG2) and a monoesterase (EstG3) to PA via their individual monoesters. In this study, whole genome sequence data were appropriately exploited using proteome and RT-PCR analyses to reveal the inducible regulation of multiple catabolic genes and operons in the assimilation of DOP isomers. Apart from the presence of a diesterase-monoesterase, the inability of strain GONU to utilize PA, MnOP and MEHP, the inability of the DnOP-induced *estG3-pht* fused operon to convert MnOP, and the involvement of NAD(P)^+^-independent dehydrogenase in the metabolism of DnOP/DEHP-hydrolysed alcohols and their dehydrogenated products are unique features of the test organism. The evaluated molecular architecture and the inducible regulation of catabolic pathways of DOP isomers achieved by gene disruption experiments have laid foundation for the purification and characterization of encoded phthalate esterases and for the development of DnOP- and DEHP-sensing whole cell bioreporters.

## Supplementary Data

Supplementary material 1
