# Supplementary material for: Evaluation of distinct molecular architectures and coordinated regulation of the catabolic pathways of oestrogenic dioctyl phthalate isomers in Gordonia sp
Source: Microbiology (Reading). 2023 Jun 29;169(6):001353. doi: 10.1099/mic.0.001353 (PMC10333787; doi:10.1099/mic.0.001353)
Supplement: Supplementary material 1 [file mic-169-1353-s001.pdf]

Supplementary Material

Evaluation of distinct molecular architectures and coordinated regulation of the catabolic pathways of estrogenic dioctyl phthalate isomers in *Gordonia* sp.

Rinita Dhar, Suman Basu, Mousumi Bhattacharyya and Tapan K. Dutta

Department of Microbiology, Bose Institute, EN-80, Sector V, Salt Lake, Kolkata – 700091, West Bengal, India

Running title: Biodegradation of dioctyl phthalate

Correspondence: Tapan K. Dutta, tapan@jcbose.ac.in

Table S1. Primers used in this study

Genes	Primer	Primer sequences (5' to 3')
**RT-PCR primers**		
estG5	Forward	CGAGGCGAACCTCTTCAAATA
	Reverse	ATCCTGGTGGTCCAGTCATA
estG2	Forward	ACCTACGTGTACCGCTATGA
	Reverse	CGTTGATGACCAGGACCTTC
estG3	Forward	GAACTTTGCAACTCAGTTGG
	Reverse	GATAGTGACGGATCAACTCG
estG1	Forward	GAAGAGCTACGACACCTTCTG
	Reverse	TCATGCCTTGTCCACCAC
estG4	Forward	GGTGAGTGTCTACGACAAGAG
	Reverse	ACGACGACGAGATCGAATG
mfst	Forward	CTTGTTGATCAGGTTTCCC
	Reverse	TACTACCGACGAGAACGG
phtB	Forward	AGTACCGCAATGAAGACG
	Reverse	GCTTGAAATTGGAGACACC
phtAa	Forward	TTCACAAGGAGGCATACG
	Reverse	GGCGTGTGGTACATGTCC
phtAb	Forward	ATGAGGCGTACCTGTTGG
	Reverse	TCCACGATGATCTCTTGC
phtAc	Forward	ATGCCCGTTGTAAAAGCC
	Reverse	TCATGCGTCCTCTACCACC
phtAd	Forward	GTCGAAATCCGTTCTTGC
	Reverse	TGAATCCCTGGAGACTGG
phtC	Forward	GCAAGCCGAAGACATCC
	Reverse	GTCGCGGATGAGTACTCC
pcaH	Forward	AGCGCATCACGATCTCC
	Reverse	AAACAGGGAGAAGTGCACG
pcaG	Forward	TTCTTCCACTACGCACTCC
	Reverse	CGTCGAGAACGAGTAGTGG
pcaB	Forward	ATTCTTTCCGTACTGGTGC
	Reverse	CGGTCTTGTCCAGCAGC
pcaC	Forward	ATCACCGATCTGACCGC
	Reverse	CAGCAACTCTTTGATCTCG
16S rRNA	Forward	ACCTGGAGAAGAAGCACC
	Reverse	CCTCAGCGTCAGTTACTACC
***estG5* gene knockout primers and restriction enzymes**		
UP-forward	*Mlu*I	CG***ACGCGT***TGTCCACACGTTGAGGAACAG
UP-reverse	*Sac*I	C***GAGCTC***GACGAACCCGGTATCTCC
DOWN-forward	*Eco*RI	CCG***GAATTC***GCGACCTGTCCACCTGGC
DOWN-reverse	*Nde*I	GGAATTC***CATATG***CGTGGGGCAGATAGGTCTCC
***estG2* gene knockout primers and restriction enzymes**		
UP-forward	*EcoRI*	*CCG****GAATTC***CTCGACACCAAGGTCACCACG
UP-reverse	*NdeI*	*GGAATTC****CATATG***CGCGGCGGTACCGAGGATGAAGG
DOWN-forward	*MluI*	*CG****ACGCGT***GTGCGGCAGCGTTCGGAC
DOWN-reverse	*SacI*	*C****GAGCTC***GGCGACAGTGCGGCCGTC

[a]Restriction recognition sequences are in italics boldface type, and enhancer sequences are underlined (single solid line).

Table S2. List of bacterial strains and plasmids used in this study

**Bacterial strains**		
	Gordonia sp.	
GONU	Wild Type, PAE degrader, kanamycin sensitive (Kan^s); Length of the wild type phthalate esterase genes are: *estG5* 1626 bp (541 aa), *estG2* 1554 bp (517 aa) and *estG3* 894 bp (297 aa). Length of wild type phthalic acid metabolizing genes are: *phtB* 867 bp (188 aa), *phtAa* 1464 bp (487 aa), *phtAb* 612 bp (203 aa), *phtAc* 198 bp (65 aa), *phtAd* 1236 bp (411 aa) and *phtC* 750 bp (249 aa). Length of wild type protocatechuic acid metabolizing genes are: *pcaH* 768 bp (255 aa), *pcaG* 570 bp (189 aa), *pcaB* 1200 bp (399 aa) and *pcaC* 1200 bp (399 aa).	This study
$GONU^{\Delta estG5}$	$EstG5^{-}Kan^r$; site directed deletion mutant of GONU *estG5*; kan^r gene cassette incorporated into the *estG5* operon by deletion of *estG5*(1296 bp from C-terminus)and deletion of intergenic space (31 bp from C-terminus)	This study
$GONU^{\Delta estG2}$	$EstG2^{-}Kan^r$; site directed deletion mutant of GONU *estG2*; kan^r gene cassette incorporated into the *estG2* operon by deletion of *estG2* (1191 bp from C-terminus), deletion of intergenic space (16 bp from C-terminus) and deletion of*orf4* (120 bp from C-terminus)	This study
	Escherichia coli	
XL1 Blue	*recA1 endA1 gyrA96 thi-1 hsdR17 supE44 relA1 lac [F′ proABlacIq ZΔM15 Tn10 (Tet^r)]*	Deb et al., 2018
**Plasmids**		
pCM184	Allelic exchange vector; Amp^r; Kan^r; Cre/Lox	Addgene<br>Deb et al., 2018
Recombinant-pCM184-*ΔestG5*	pCM184 harboring*estG5* DOWN fragment in MCS1 and *estG5* UP fragment in MCS2; Amp^r; Kan^r; Cre/Lox	This study
Recombinant-pCM184-*ΔestG2*	pCM184 harboring*estG2*UP fragment in MCS1 and *estG2*DOWN fragment in MCS2; Amp^r;Kan^r; Cre/Lox	This study

Table S3. Oxygen uptake rates[a] with various compounds by resting cell transformation of *ΔestG5* and *ΔestG2* mutants of *Gordonia* sp. strain GONU grown on succinate-DnOP and succinate-DEHP, respectively.

Substrate	Oxygen uptake rate by *ΔestG5* mutant cells grown on[b]:	Substrate	Oxygen uptake rate by *ΔestG2* mutant cells grown on[b]:
	**Succinate-DnOP**		**Succinate-DEHP**
DnOP	ND	DEHP	ND
MnOP	ND	MEHP	49.73
PA	38.73	PA	35.46
PCA	ND	PCA	ND
1-Octanol	0.83	2-Ethyl-1-hexanol	ND
1-Octanal	0.66	2-Ethyl-1-hexanal	0.19
1-Octanoic acid	ND	2-Ethyl-1-hexanoic acid	ND

[a]All values are corrected for endogenous O_2 uptake. ND, Not detected.

[b]Oxygen uptake rate in (nmol O_2 consumed) min^{-1}(mg of protein)$^{-1}$

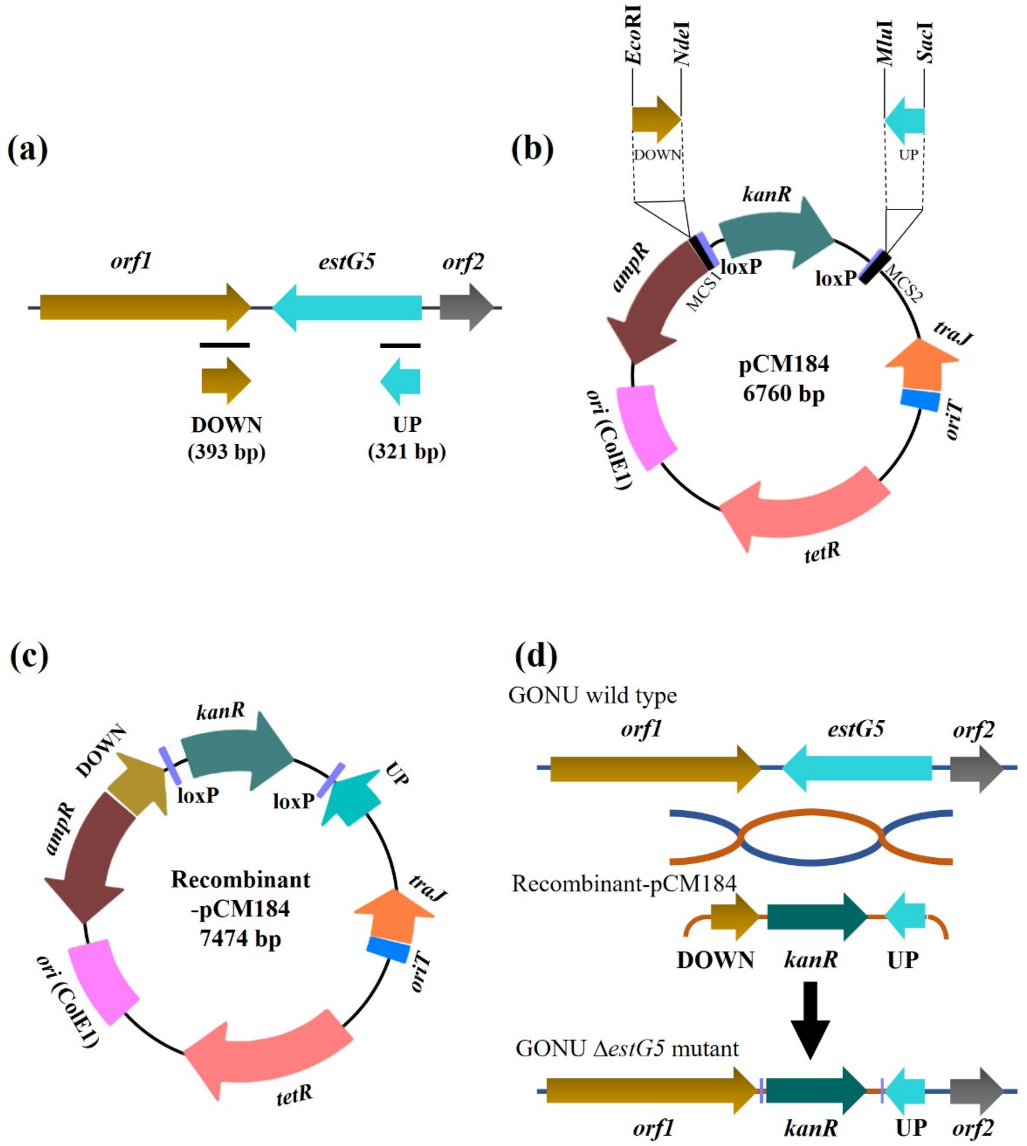

Fig. S1. Construction of *ΔestG5* mutant of *Gordonia* sp. strain GONU. (a) Genetic organization of *estG5* involved in the hydrolysis of DnOP. Black bars represent the genomic positions of the amplified UP and DOWN fragments used to construct the recombination plasmid. pCM184 used as the vector backbone (b) where the UP and DOWN fragments were cloned in the depicted orientation to construct the recombinant-pCM184 (c). Chromosomal insertion of *kanamycin* cassette with the disruption of *estG5*, occurred via homologous recombination of the recombinant pCM184 and GONU chromosome in the construction of the *ΔestG5* mutant strain (d).

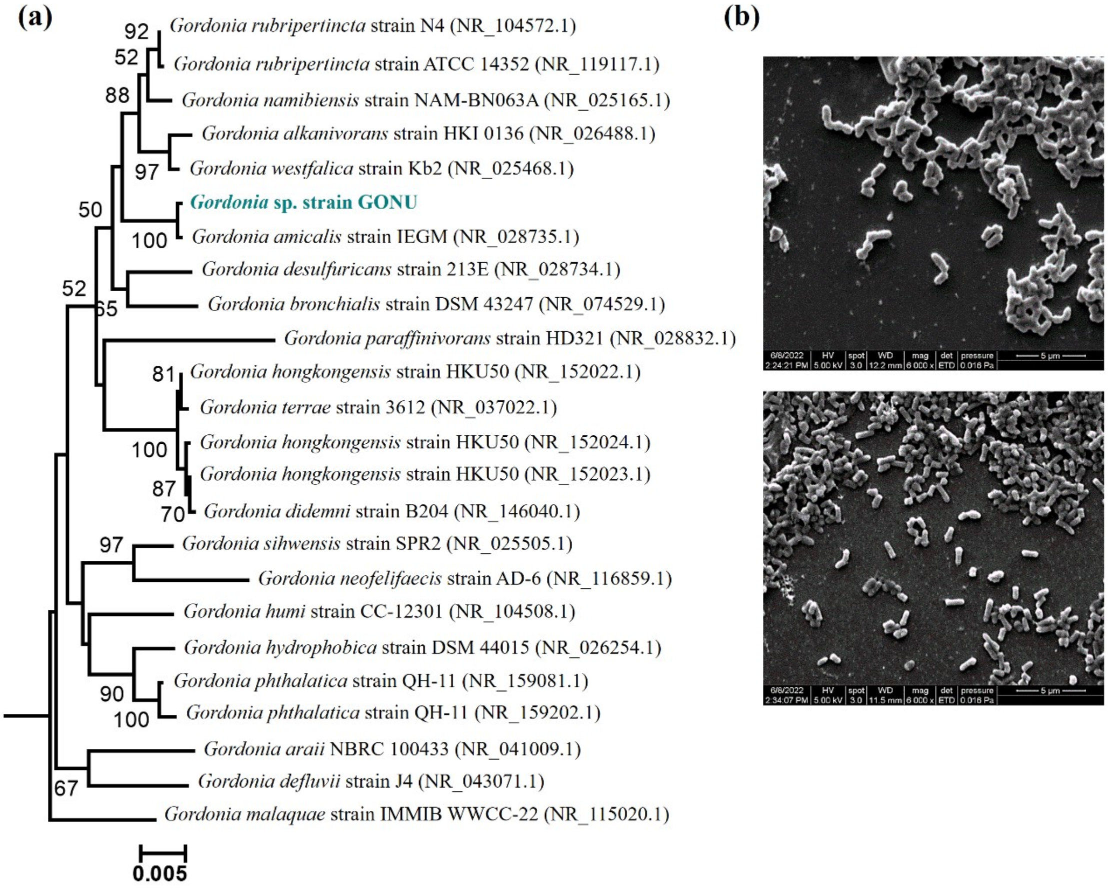

Fig. S2. (a) Phylogenetic relationship of strain GONU and representative strains of the genus *Gordonia* based on 16S rRNA gene sequences. Numbers at the nodes indicate the levels of bootstrap support based on neighbor-joining analysis of 100 resampled data sets. Bootstrap values below 50% are not shown. The scale bar represents 0.005 substitutions per nucleotide position. The GenBank accession numbers of the sequences are indicated within parentheses. The multiple sequence alignment was performed using ClustalX2 and the phylogenetic tree was constructed using neighbor-joining algorithm as implemented in Tree Explorer 2.12. Strain GONU is marked in colored boldface. (b) The scanning electron microscopic images at 6000× magnification of log-phase cells of strain GONU grown in the presence of succinate (top) and DnOP (bottom).

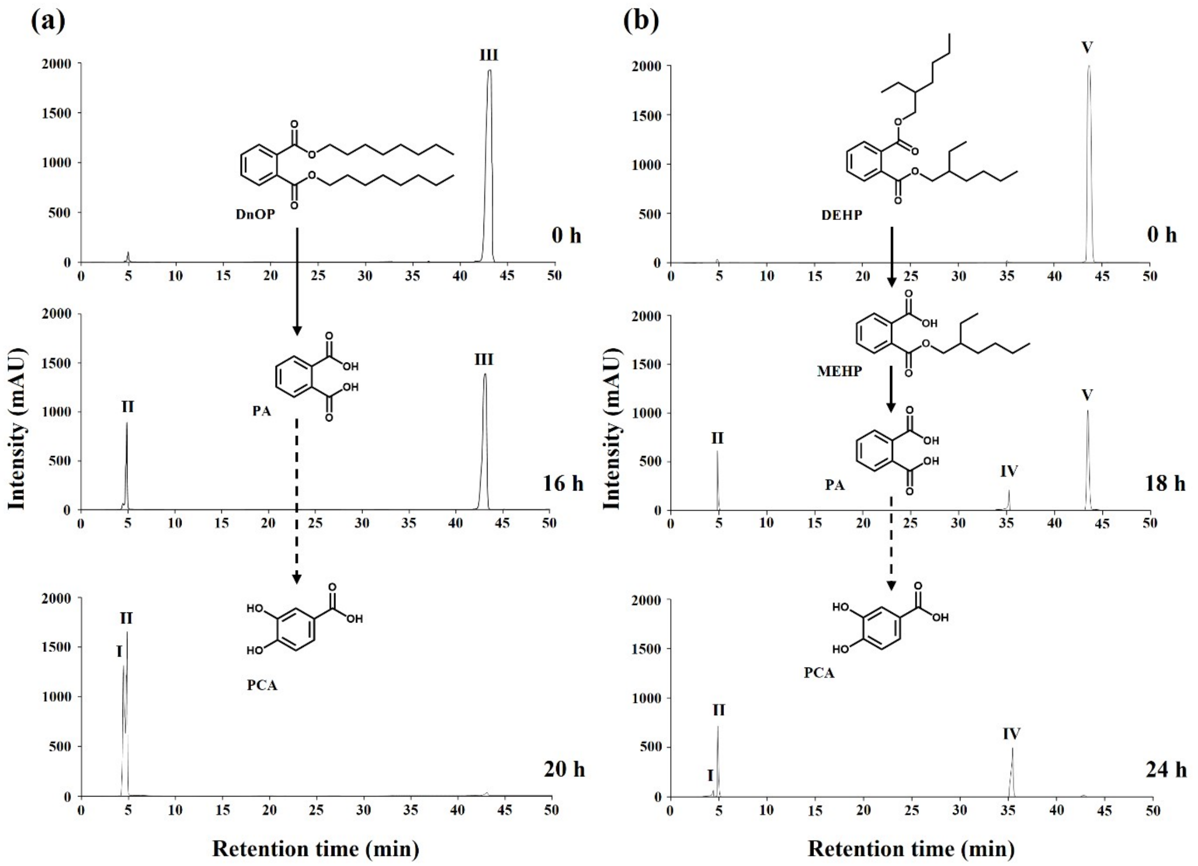

Fig. S3. Identification of metabolites by HPLC analysis. (a) Formation of phthalic acid (PA, peak II) and protocatechuic acid (PCA, peak I) from di-*n*-octyl phthalate (DnOP, peak III) by DnOP-grown cells of strain GONU incubated for 0 h, 16 h and 20 h. (b) Formation of mono-2-ethylhexyl phthalate (MEHP, peak IV), PA and PCA from di(2-ethylhexyl) phthalate (DEHP, peak V) by DEHP-grown cells of strain GONU incubated for 0 h, 18 h and 24 h.

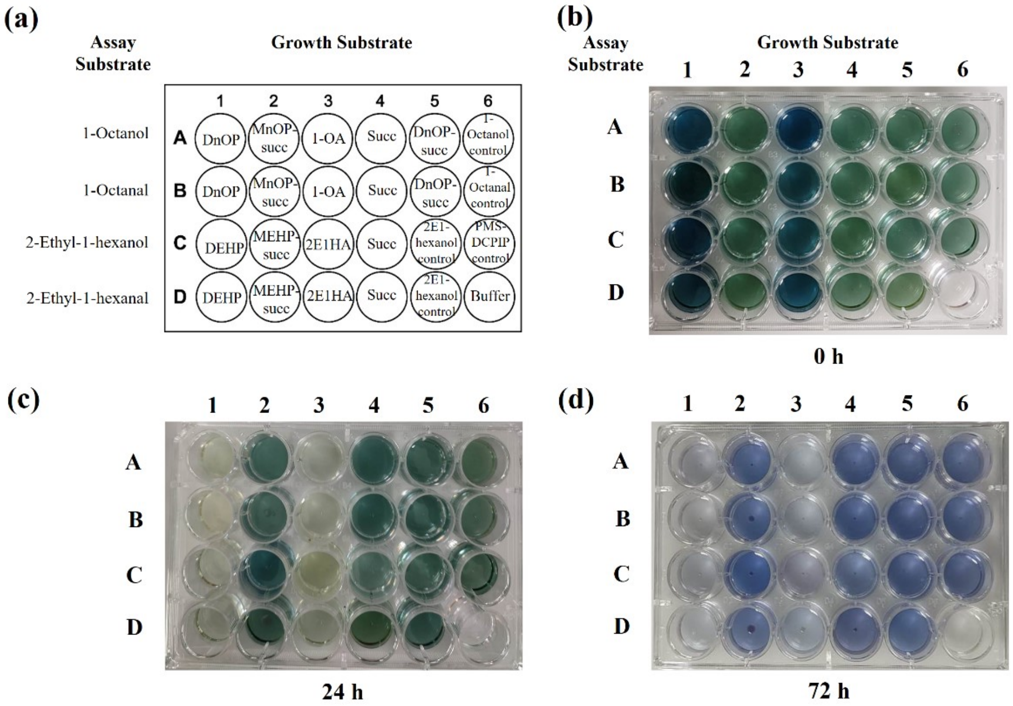

Fig. S4. DCPIP reduction assay for the evaluation of $NAD(P)^+$-independent alcohol and aldehyde dehydrogenase activities. The cell-free extracts (CFEs) of cells of wild-type and mutant (*ΔestG5*) strains grown separately in the presence of DnOP, DEHP and their metabolic intermediate or succinate were used for the analysis, where 1-octanol, 1-octanal, 2-ethyl-1-hexanol and 2-ethyl-1-hexanal were used as the assay substrates, so depicted in the layout of a microtitre plate (a). The lane A and B of column 5 contain mutant (*ΔestG5*) strains, grown on DnOP + succinate. Changes in color are observed following incubation of reaction mixtures for 0 h (b), 24 h (c), and 72 h (d). The CFE of succinate-grown cells was used as negative control, and 50 µg CFE was used for each reaction.

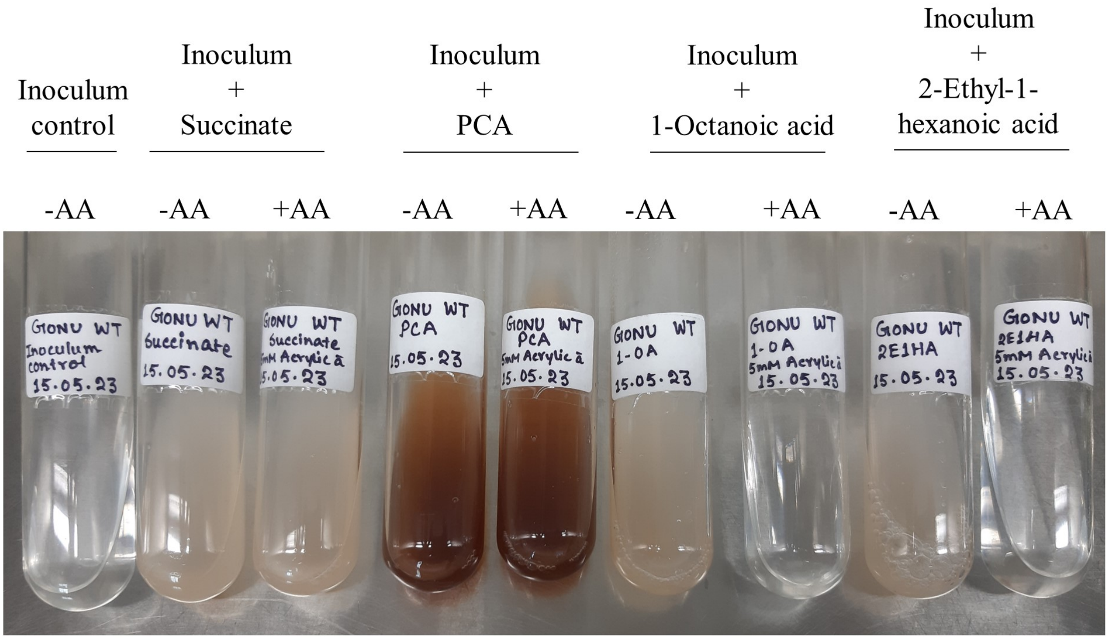

Fig. S5. β-Oxidation inhibition assay using acrylic acid. The panel represents the growth profile of wild-type cells in different substrates supplemented with or without acrylic acid (5 mM). Succinate and protocatechuic acid (PCA)-grown cells were kept as positive controls, where growth was detected in the presence of 5 mM acrylic acid. No growth was observed in the presence of 1-octanoic acid or 2-ethyl-1-hexanoic acid supplemented with acrylic acid.

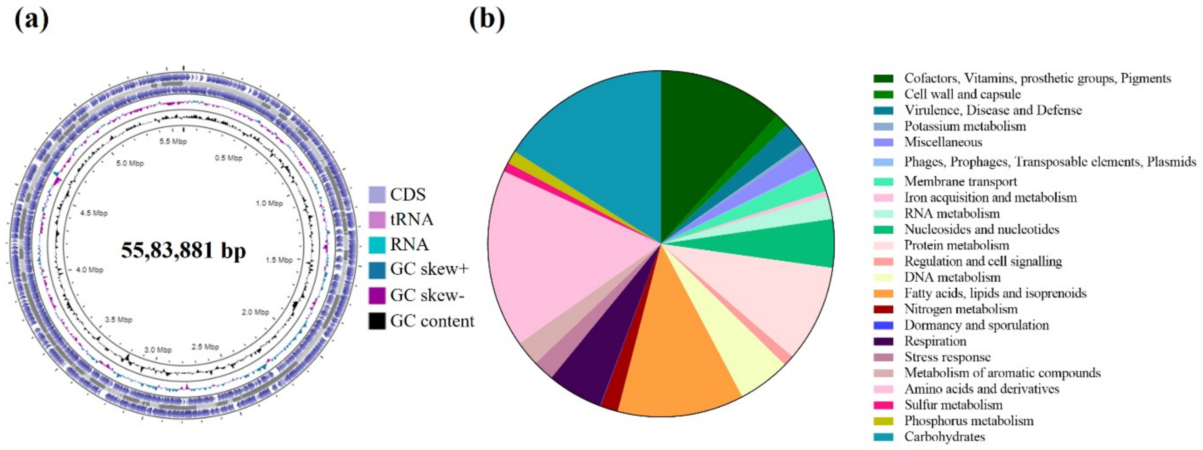

Fig. S6. (a) Circular visualization of draft genome of *Gordonia* sp. strain GONU. (b) COG functional classification of encoded proteins in the genome.

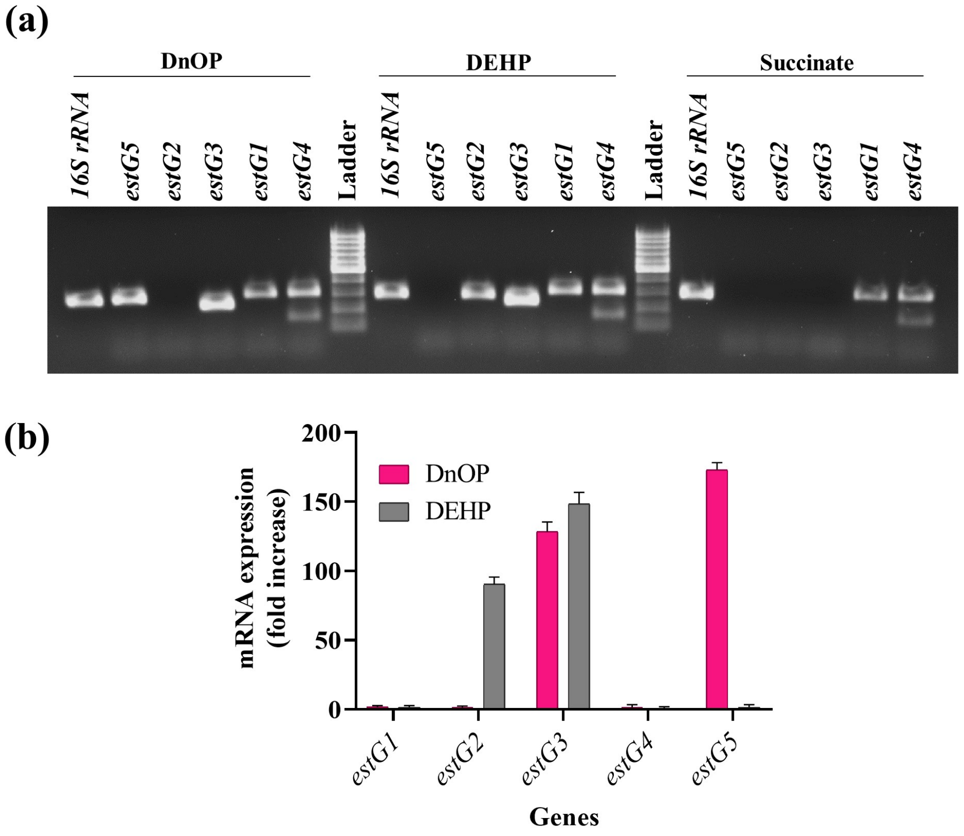

Fig. S7. RT-PCR analysis of the mRNA transcripts of five esterase/hydrolase genes. (a) Agarose gel electropherogram profile showing relative changes in gene expression. (b) Bar graph represents fold change of the transcripts of the catabolic genes, quantified by RT-qPCR. The succinate-grown cells were taken as the negative control, while *16S rRNA* was kept as the endogenous control. Mean values were obtained from triplicate measurements. The esterases/hydrolases were selected for analysis based on their upregulated profiles in the proteomic study.

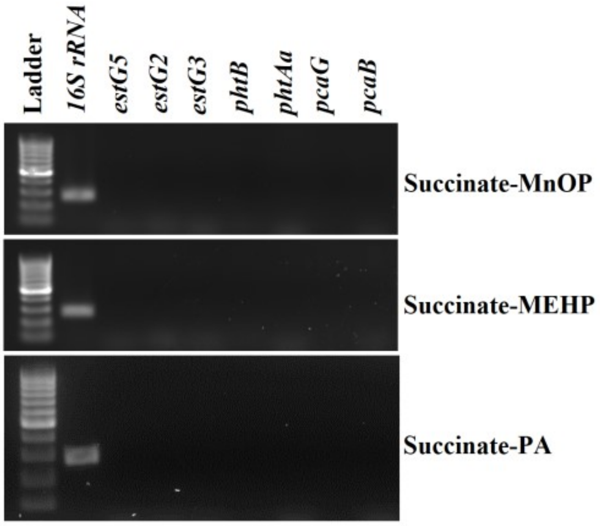

Fig. S8. Agarose gel electropherogram profile of the RT-PCR analysis of mRNA transcripts of *est*, *pht* and *pca* genes of the cells of strain GONU, grown on succinate in the presence of MnOP, MEHP or PA. *16S rRNA* gene was used as a positive control.

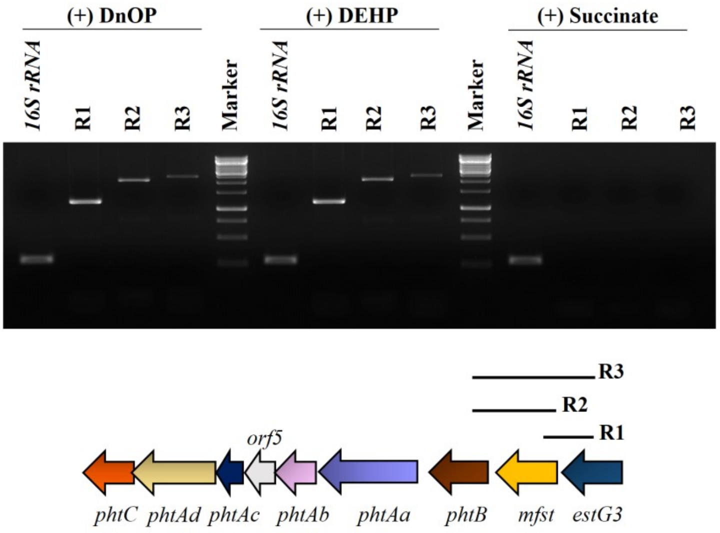

Fig. S9. Agarose gel electropherogram profile showing end-point PCR analysis of mRNA transcripts of different intergenic regions of *estG3-pht* operon in the DnOP, DEHP and succinate-grown cells of strain GONU. R1 (*estG3* – *mfst* fragment), R2 (*mfst* – *phtB* fragment) and R3 (*estG3* – *phtB* fragment). *16S rRNA* gene was kept as endogenous control.

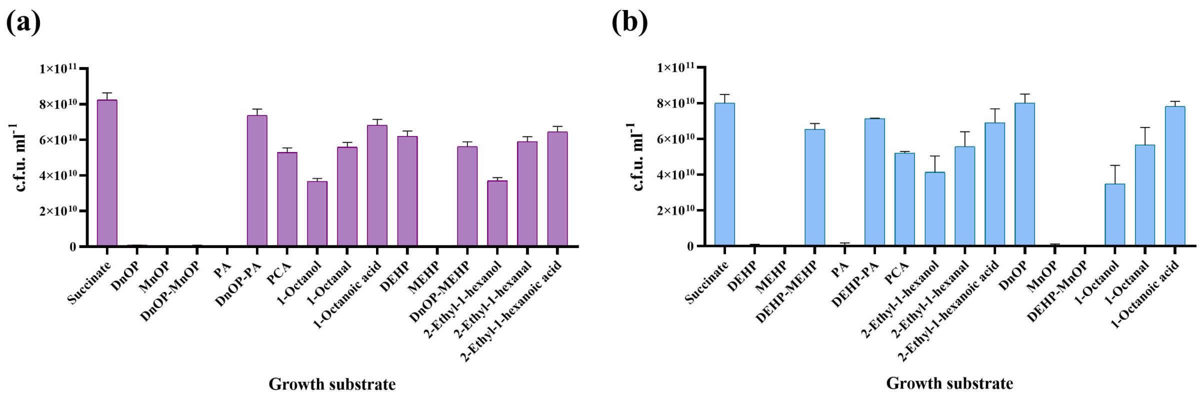

Fig. S10. The bar graphs represent the growth of (a) *ΔestG5* mutant and (b) *ΔestG2* mutant in minimal salt medium supplemented with different substrates and metabolic intermediates upon incubation for 30 h at 28°C.

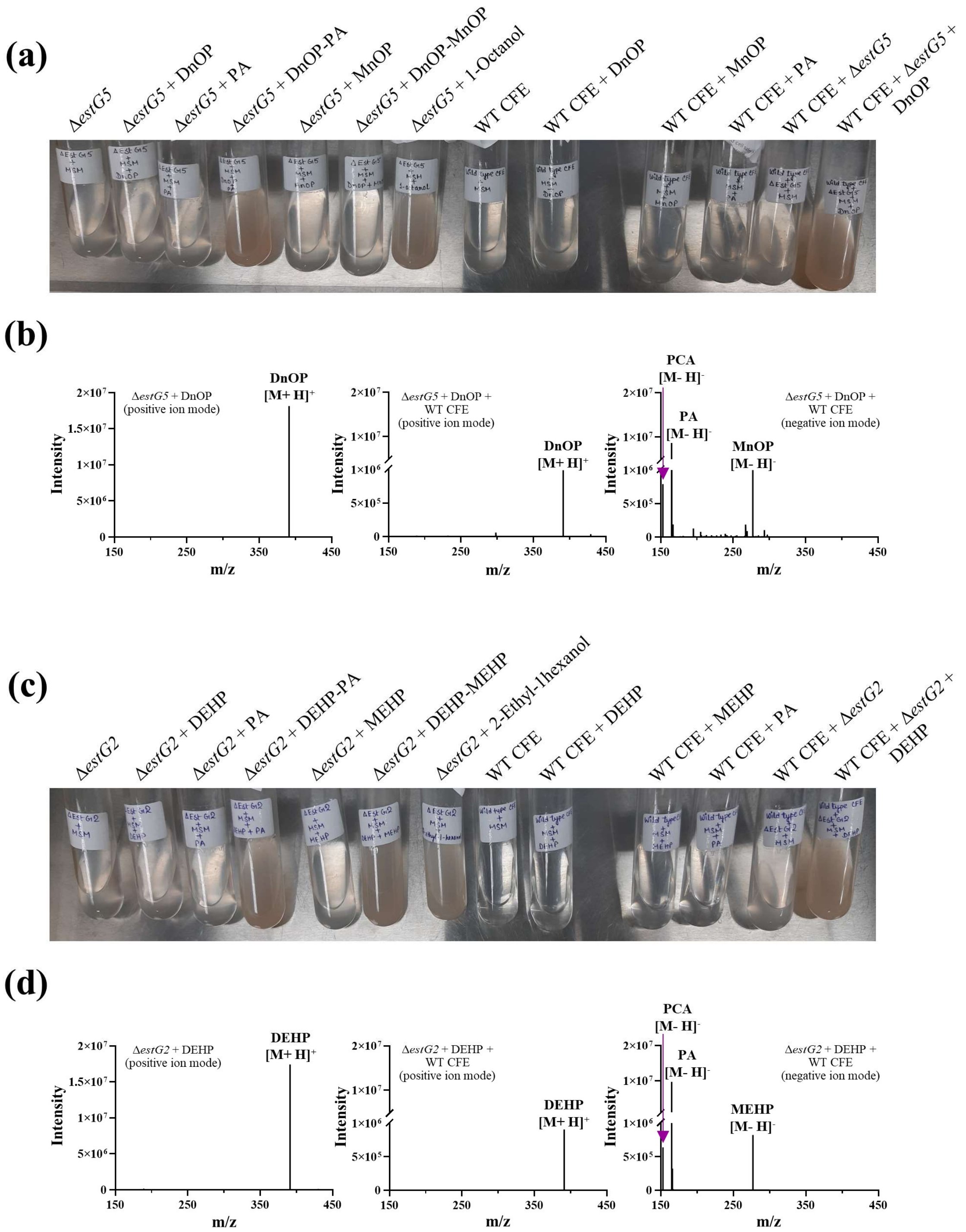

Fig. S11. The roles of EstG5 and EstG2 and the phenotypes of the *ΔestG5* and *ΔestG2* mutants. The panel (a) represents the incubation profile of Δ*estG5* mutant with DnOP and its metabolic intermediates in the presence and absence of the cell-free extract (CFE) of DnOP-grown cells of wild-type (WT) strain. The Panel (b, from left to right) represents the DI-ESI-MS analysis of the products obtained from the incubation of Δ*estG5* mutant with DnOP in the

absence and in the presence of the CFE of DnOP-grown cells of WT strain under positive ion $[H]^+$ mode and/or negative ion $[H]^-$ mode. The panel (c) represents the incubation profile of Δ*estG2* mutant with DEHP and its metabolic intermediates in the presence and absence of the CFE of DEHP-grown cells of WT strain. The Panel (d, from left to right) represents the DI-ESI-MS analysis of the products obtained from the incubation of Δ*estG2* mutant with DEHP in the absence and in the presence of the CFE of DEHP-grown cells of WT strain under positive ion $[H]^+$ mode and/or negative ion $[H]^-$ mode.

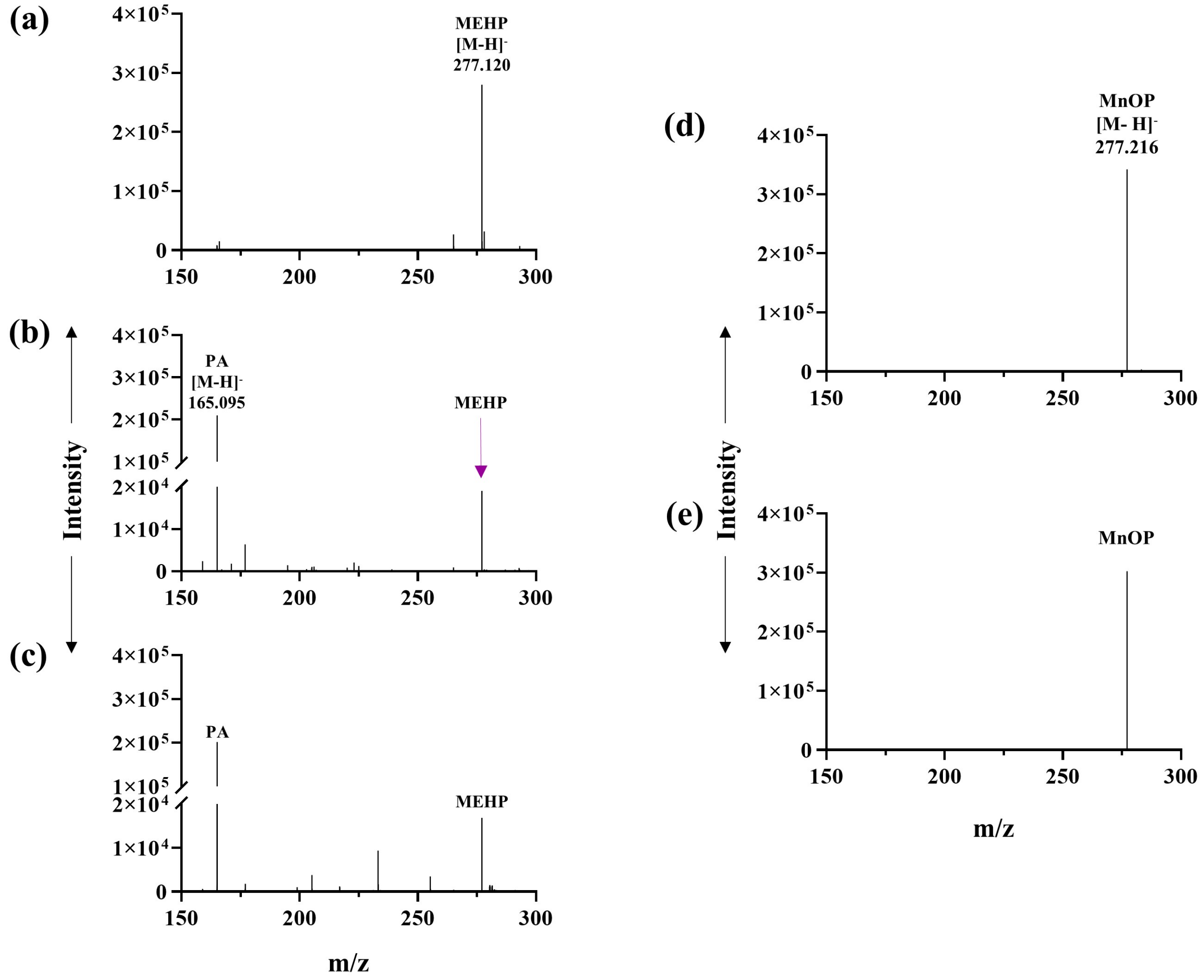

Fig. S12. DI-ESI-MS analysis of cell-free extract-mediated transformation of MEHP and MnOP in strain GONU. The figure represents (a) MEHP (0.5 g l^{-1}) as substrate control and the conversion of MEHP to PA in (b) wild type DnOP-grown cells and (c) DnOP-induced *ΔestG5* mutant cells. In addition, the data shows (d) MnOP (0.5 g l^{-1}) as substrate control and (e) MnOP conversion profile in wild-type DEHP grown cells. All the analyses were made in

negative ion mode $[M-H]^-$. The enzymatic reactions were conducted at 28°C for 1 h using 100 μg of crude protein.
